# Transformative social innovation and rural collaborative workspace assemblages as a means of prefiguring community economies

**DOI:** 10.12688/openreseurope.18007.1

**Published:** 2024-09-19

**Authors:** Colm Stockdale, Vasilis Avdikos

**Affiliations:** 1 Department of Economic and Regional Development,, Panteion University of Social and Political Sciences, Athens, L. Syggrou 136, 16761, Greece

**Keywords:** Collaborative Workspaces, Social Innovation, Diverse Economies, Post Capitalism, Assemblage, Rural

## Abstract

**Background:**

Collaborative Workspaces are rapidly growing and evolving across the world. Traditionally understood as an urban phenomenon, most research understands them as either ‘entrepreneurial-led’, as profit-driven and commercial spaces such as business incubators and accelerators, or ‘community-led’ as being bottom-up, not-for-profit ventures aimed at catering for the needs of their community. Recent years however have seen their diffusion beyond large urban agglomerations to small towns and villages, with their functions assumed to be more community-orientated. At the same time, social innovation, or social innovation processes have been gaining prominence in academia, policy, and practice, as they address societal problems and hold potential for new forms of social relations. This paper attempts to provide a novel framework towards understanding the transformative potential of rural collaborative workspaces, as they engage in processes of social innovation, by drawing from diverse and community economies literature and assemblage thinking.

**Methods:**

The paper uses international case study comparison between rural Austria and Greece (One case from each country). Methods applied were: semi-structured interviews (N=28), participant observation and focus groups (2).

**Results:**

Community-led rural collaborative workspaces hold transformative potential from i) their ability to assist rural actors with their capacities and realizing their desires and ii) changing individual subjectivities towards collective. Through changing social relations in praxis and perceptions, we examine how social innovation processes through collaborative workspaces can be understood as a means of opening new economic subjectivities towards creating community economies as their transformative potential.

**Conclusions:**

Although rural collaborative workspaces hold potential for societal transformation, they require further institutionalization and support to move beyond the interstitial and symbiotic stages of transformation.

## Plain language summary

This article aims to understand how collaborative workspaces, also known as hubs, have the potential to transition away from capitalism, by valuing other practices that hold greater social and environmental considerations.

## Introduction

Collaborative workspaces (hereafter CWS) have emerged initially in urban areas since the mid-2000s. Coworking spaces, creative hubs, hackerspaces, fab labs etc host nowadays more than 5 million coworkers worldwide (ergonomictrends.com). Although primarily an urban phenomenon, CWS have also started to spread in medium and small cities in the last few years, away from urban agglomerations ( Avdikos & Merkel, 2020). Recent research suggests that 66% of CWS are located in urban regions, while the rest (34%) are in intermediate or rural regions ( Marmo & Avdikos, 2024). CWS usually provide workspaces to freelancers, paid employees, digital nomads and remote workers in general, but also, they facilitate professional encounters and networking possibilities between different people and they can also act as mediums for cultural exchanges. Especially, CWS in small cities and towns can play the role of a socio-cultural center where locals can engage through new participatory modes in different kinds of projects that benefit their localities and that can eventually end up in social innovation (hereafter SI) processes and projects. The paper aims in unpacking the role of CWS as initiators and facilitators of SI processes in rural areas, looking at two community led CWS in Western Greece and Upper Austria.

According to Moulaert *et al.* (2017) SI is i) a way of understanding a wide range of activities and practices oriented to addressing and *providing solutions to specific social problems* or meeting human needs. Moreover, SI ii) involves *changing relations* through the adoption of new social practices, institutional arrangements and/or forms of participation. This means that SI does not separate means from ends, but treats needs and problems as inherent in social relations. However, the effects of SI extend beyond the immediate meeting of needs. SI seems to improve long term opportunities for individuals and communities, through producing more efficient, effective and sustainable means for society to deal with its challenges, and that iii) can have a deeper *transformative impact* over communities and society.

Through the analysis of qualitative data, mainly through interviews and participant observation, we delve into the multiple sets of relations found in these two rural CWS and attempt to unpack the ways these arrangements cover local needs and at the same time they foster new social relations. Moreover, we engage with the literature in unpacking the ways CWS can also cause a transformative effect on social relations towards a post growth/post-capitalist society, challenging the ways local institutions work. To do so, a novel theoretical framework, consisting of Diverse and Community Economies and Assemblage Thinking will be elaborated upon.

## Social innovation, assemblages and diverse economies: a literature review

The use of SI over the last 30 years has increased almost exponentially ( Van der Have & Rubalcaba, 2016). Within academia the term’s meaning varies across fields such as innovation studies, management studies, sustainable and territorial development ( Howaldt *et al.*, 2014). This wide array of understandings is said to both be of help ( Moulaert & MacCallum, 2019) and hinderance ( Bock, 2012) to understanding the ‘fuzzy’ concept that is SI (ibid.). While not providing an exhaustive literature review of SI, this section will serve to unravel SI, while positioning the paper within the literature and allowing for a reconceptualization of the concept through Diverse and Community Economies ( Gibson-Graham, 2008).

SI seems a ‘contested’ concept ( Ayob *et al.*, 2016; Marques *et al.*, 2018). On the one hand ‘social’ innovation can be understood as social entrepreneurship whereby social entrepreneurs come up with innovative ideas to address society’s most pressing challenges, while the other approach is more understood through collective action and the social and solidarity economy. We want to highlight the capitalocentric nature ( Gibson-Graham, 2008) of the entrepreneurial understandings of SI, and aim to demonstrate how SI processes work beyond an organization, or individual entrepreneur. While not reducing the role they can have in these processes, we argue that SI is also not reducible to one actor, but it operates as a SI assemblage, as a whole of different actors that have a collective desire in addressing social problems.

Within the capitalocentric strand, SI is understood as a technocratic, entrepreneurial/market driven complement to the neoliberal state (e.g. Mulgan, 2006; Murray *et al.*, 2010). Much of the focus is on individual entrepreneurship and mainstream innovation approaches to ‘solving’ society’s most pressing problems. Here, SI is understood as ‘ *an idea of a need that isn’t being met, coupled with an idea of how it could be met’* ( Mulgan, 2006: 149) or more specifically “ *new ideas (products, services and models) that simultaneously meet social needs and create new social relationships or collaborations. In other words, they are innovations that are both good for society and enhance society’s capacity to act.”* ( Murray *et al.*, 2010: 3). Such understandings promote a capitalocentric worldview, whereby civil society is viewed as an untapped source of innovative capacity that can be harnessed towards creating new markets and economic value. Such understandings have also pierced policy documents, with SI seen allowing governments to ‘do more with less’ ( BEPA, 2014), increasing growth and competition while addressing society’s most pressing problems ( Fougère *et al.*, 2017).

Several scholars critique this capitalocentric discourse, generally as it proliferates both neoliberal governance and subject production ( Bragaglia, 2021; Fougère *et al.*, 2017; Jessop *et al.*, 2013; Swyngedouw, 2005). In these interpretations, SI is understood as quick-fix solutions to social problems through the creation of active economical and entrepreneurial subjects for a capitalist economy ( Arampatzi, 2022; Fougère *et al.*, 2017; Jessop *et al.*, 2013), while shifting social and environmental costs from the state to citizens ( Fougère *et al.*, 2017; Swyngedouw, 2005). 

Moving to the more critical strand of the literature, it has been developed over the last 30 years by Frank Moulaert and colleagues (e.g. Moulaert *et al.*, 2010; Moulaert *et al.*, 2013), who developed SI as a concept vis-à-vis neoliberal practices and policies, originally understood as an alternative model of local development ( Moulaert *et al.*, 2005). With its roots in new social movements ( Godin, 2012; Martinelli, 2010), SI is understood not as an organizational form, but as ethical spatial projects, by changing social relations towards meeting human needs and not as a gap in the market to be addressed. Moulaert & Mehmood (2020: 17), portray how many SI’s are rooted in socio-ecological movements, as consequences of increasing privatization, climate crisis and increasing social inequalities. Examples include the new-municipalist movements (e.g. in Madrid and Barcelona), the creation of new urban and rural commons, agroecological initiatives, local development experiments aiming to move past the market-based economy etc.

Thus, SI is broadly understood as *innovation in social relations by meeting human needs* ( Moulaert *et al.*, 2013). SI is understood as both a practice (collective satisfaction of human needs) and a process (changes in social relations, collective empowerment) ( Moulaert & Mehmood, 2020). Part of our contribution is through understanding the underlying processes that lead to collective action, SI can be understood as a means of opening new economic subjectivities and post-capitalist possibilities ( Gibson-Graham, 2008; Wright, 2010) which we would like to conceptualize as the transformative potential of SI, building through Assemblage thinking, as the analytical tool.

### Transformative SI

Despite the split in SI literature, there is a nascent literature regarding SI and societal transformation, that transcends the binary ( Moulaert & MacCallum, 2019). An important contribution to its theory has been that of the EU funded TRANSIT project to develop a theory on transformative social innovation (e.g. Avelino *et al.*, 2019; Haxeltine *et al.*, 2016; Pel *et al.*, 2020). Here, transformative SI is understood as a “ *process, through which social innovations challenge, alter and/or replace established (and/or dominant) institutions in the social-material context*” ( Haxeltine *et al.*, 2016: 19). The theoretical framework of the project comes from sustainability transitions thinking and conceptualizes transformative SI through the Multi-Level Perspective framework (MLP) ( Geels, 2004), whereby SI’s need to adapt to dominant rule sets in society and adapt to institutional logics (such as state regulations or collaborating with institutions) to become institutionalized and have a transformative impact in society ( Pel *et al.*, 2020). Despite the value however, there are several conceptual limitations on this approach, that are generally existent in most transitions’ literature. Firstly, there is a lack of critique regarding capitalism ( Chatterton, 2016; Törnberg, 2021) and secondly the MLP privileges structure and hierarchy, reproducing the very power relations responsible for the need for social change to take place ( Gillard *et al.*, 2016).

In contrast, coming from an anti-essentialist and relational ontology inspired by Deleuze and Guattari and Gibson-Graham, we aim to overcome such limitations. Gibson-Graham & Roelvink (2009) highlight how a re-reading of the three dimensions of SI (addressing social needs (through the inclusion of marginalized groups into society), changing social relations and collective empowerment/socio-political transformation) through Diverse and Community Economies (hereafter DCE) can enrich SI theory. Through a re-conceptualization of the economy as a diverse space of ethical and political action “what is usually regarded as ‘the economy’ —wage labor, market exchange of commodities, and capitalist enterprise” ( Gibson-Graham, 2008: 69), becomes “just one particular set of economic relations situated in a vast sea of economic activity” ( Gibson-Graham, 2008: 70). Rather than a dominant capitalist economy, Gibson-Graham (2008) ask us to read for difference over dominance, and understand the diversity of economic activity at play, such as “non-market gifts, volunteer work, commons, cooperative forms of production, criminal economies and much more” ( Smith, 2020: 596).

Gibson-Graham & Roelvink (2009) use the example of Argentina post-2008 financial crisis, to demonstrate how SI processes can encourage alternative modes of development, with interstitial transformations emerging from the cracks of a crisis stemming from the capitalist economy – “ *the unemployed started to build community economies by engaging in barter and using alternative currencies, providing neighbourhood-based social services and schooling, and taking over factories and running them cooperatively”* (P. 32). What is key to these intentional/community economies was the processes that preceded them – “ *To transform themselves into community economic subjects, they created a cooperative radio station; they went to the World Social Forum in Porto Alegre to see themselves reflected in others who were also engaged in projects of self-determination.”* (p. 32). It is this process of subject formation for the community economy and opening up of economic diversity in the economy we would like to stress as the desired transformation for post-capitalism, without stressing an end product, but process, struggle and deliberation ( Gibson-Graham & Roelvink, 2009).

Furthermore, in *Real Utopias* Olin Wright (2010) develops a robust theoretical framework for post-capitalist transformation through envisaging real utopias - *‘proposals for pragmatically improving our institutions. Instead of indulging in utopian dreams we must accommodate to practical realities.’ (p. 4).* Wright identifies three main ‘pathways’ for social transformation: radical ‘ruptural’ transformation through wide-scale revolt against state and market, while bringing in radical alternatives; ‘symbiotic’ transformation is through long term co-operation with existing structures and institutions (e.g. trade unions); while ‘interstitial’ transformations take a prefigurative approach, by creating future alternatives in the here and now. Regarding SI and post-capitalist transformations, interstitial transformations aid us in their conceptualization, by seeking to create new forms of social empowerment within the niches and margins of capitalism, while building social power through civil society, they mirror many features of the grassroots conceptions of SI.

To give these re-conceptualizations of SI further analytical power, we draw on Assemblage thinking ( Deleuze & Guattari, 1980). The main contribution of assemblage is analysing socio-spatial phenomena and understanding them relationally as dynamic wholes, in constant flux, or becoming. Rather than their essential qualities, it gives priority to relations and qualities, enabling a mode of analysis where we can compare bodies through their given affective relations, ( Buchanan, 1997). The term ‘assemblage’ is a translation from the French - *agencement* - originally applied by Deleuze and Guattari to signify the capacity to act. Agencement holds more meaning than the English ‘assemblage’, including to arrange, to dispose to fit up, to combine, to order ( Law, 2004: 41). Therefore, while assemblages are made up of material, expressive and symbolic components, we must look at them beyond a collection of things but their interaction, arrangement, ordering and (dis)assembly. Moreover, we must look beyond the components themselves, which are just the “props needed to actualize a particular arrangement of desire.” ( Buchanan, 1997: 65), thus assemblages are desire driven.

Following other approaches drawing from Deleuze & Guattari in trying to understand SI, we want to highlight the rhizomatic nature of social relations ( Pel *et al.*, 2020; Scott-Cato & Hillier, 2010). Rather than essentializing SI processes, we view changing social relations as a non-linear, rhizomatic process that occurs through the culmination of everyday actions, without a necessary beginning or end. Rhizomatic change is a micropolitics of becoming, that seeks to open new possibilities and multiplicities, towards post-capitalist futures. We want to “ *focus on process rather than on product, on struggle and deliberation rather than on an image of a predefined collective identity or geographical locality.”* ( Gibson-Graham *et al.*, 2020: 5).

Moreover, throughout the analysis we will draw heavily from the concept of affect, understood as the capacity to affect and be affected. When bodies enter affective relations with one another, it refers to those ‘encounters’ that may augment, or diminish their respective capacities ( Slaby & Mühlhoff, 2019). This is central to our assemblage analysis, as we will demonstrate how various (human and non-human, material and expressive) component parts affect the assemblage and its capacities. This autonomy of parts can also be referred to as the exteriority of relations ( DeLanda, 2006). Affective labour ( Hardt, 1999) understood as the production and manipulation of affects, will also be essential to our understanding of the CWS role in organizing social relations, and subject formation, as individual capacities are made, unmade and re-made ( Slaby & Mühlhoff, 2019).

## CWS

CWS refers to spaces, which are primarily work purposed environments, but they can also be places where people come together for both work and other personal activities in a shared space such as coworking spaces, makerspaces, fablabs, business incubators and accelerators, social and cultural centres etc. ( Schmidt & Brinks, 2017). CWS have been described as sites of ambivalence, demonstrating tensions between both corporate and counter-corporate, capitalistic and commoning, collaborative and individualistic ways of being ( Bandinelli & Arvidsson, 2013; De Peuter *et al.*, 2017; Vidaillet & Bousalham, 2020). This contrast displays the plurality of CWS, the term may relate to work collectives, co-operatives for freelancers and other forms in response to precarious markets, which operate with values of the social and solidarity economy; or alternatively CWS may act as business incubators or accelerators for start-ups, along with corporate chains of coworking ( Arvidsson, 2020; Brooke *et al.*, 2014; De Peuter *et al.*, 2017). Contributions generally differentiate between entrepreneurial-led vs community-led CWS ( Avdikos & Iliopoulou, 2019; Avdikos & Merkel, 2020), with the latter also referred to as resilient CWS ( Gandini & Cossu, 2021). While both types overlap in their activities and functions ( Avdikos & Pettas, 2021), community led CWS generally are not-for-profit, and have an authentic concern for their community ( Avdikos & Iliopoulou, 2019).

Moreover, there is a growing number of contributions referring to the political economy of CWS, and their potential for fostering alternative economic practices (e.g. Smith (2020); Vidaillet & Bousalham, 2020. While not always offering a complete alternative to the market, it appears that a number of community led CWS may oscillate between the market and the commons ( Avdikos & Pettas, 2021). For example, while CWS may rent out office space for freelancers, they may simultaneously offer their space to be used by the local community (e.g. neighbourhood associations) for events and other services ( Brown, 2017).

More recently, the CWS phenomena has diffused to rural and peripheral areas, yet questions remain about how their dynamics may both compare and contrast to their urban counterparts. Multiple sources (e.g. Görmar, 2021; Tomaz *et al.*, 2022) have highlighted that CWS in rural areas are more diverse in the activities they offer than urban CWS, ranging from community services to social and cultural initiatives. Through offering a range of services, such CWS can act as a ‘social hub’, or ‘social infrastructure of care’ for communities, by both creating and maintaining the social fabric of rural areas ( Avdikos & Merkel, 2020; Marmo & Avdikos, 2024; Merkel, 2023).

Community-led CWS can facilitate grassroots innovation practices when used by local citizens to address local social needs ( Dias & Smith, 2018). These CWS thus can be used as a tool to foster economic diversity ( Gibson-Graham, 2008; Smith, 2020) by engaging in simultaneous capitalist, alternative and non-capitalist practices, and they can act as ‘syntopias’ for their communities ( Vidaillet & Bousalham, 2020).

## Methods

The research applied a qualitative case study approach ( Yin, 2018). Data collection was carried out for roughly one month in each case. between March to June 2023. A key informant (the manager of the CWS) was used to gain access to the CWS, local people and various activities and events that took place during the stay. The study applied qualitative research methods: i) participatory observation, ii) semi-structured interviews, and iii) focus groups. The researcher engaged in a total of over 3,000 hours of participant observation, with the role changing throughout the data collection period, from a ‘participating observer’, ‘partially participating observer’ and ‘minimally participating observer ( Bryman, 2012), depending on the situation. Observations were supported by 30 semi-structured interviews as a means of understanding how individuals experience and make sense of their own lives. Interviews were conducted with those directly involved with the CWS (e.g. managers, workers, volunteers, individuals and groups who use the services, such as institutional partners, and those indirectly involved with the CWS (e.g. locals who do not use the CWS). Prior to fieldwork, a research proposal was submitted and approved by the ethics committee at Panteion University. (Protocol Number: 44/ 30-9-2022). In line with ethics guidelines, the names of the interview participants and CWS have pseudonymized to protect the identities of those who participated in the study.

### Case studies

For reasons of anonymity, we call the Greek case Western Greece Hub (WGH) and Austrian case Collaborate Upper Austria (CUA). While research took place in two small towns in the regions of Western Greece and Upper Austria, just the regions will be referred to. WGH is a non-profit organization founded as a legal entity in 2019. The organization emerged from a former local activist group concerned about the downward trajectory of the town. Their purpose is to strengthen the collectivity in the local community through the participatory mapping of the city and its culture, preserving its environmental wealth and the creation of programs of alternative cultural tourism and ecotourism. Since 2019 the organization has a ‘Local Hub’ which acts as a node for community activities of the town. Among their many activities include a gift shop with local products, boat tours, art classes, creative activities for children, providing a meeting point for groups, Info Point, an annual cultural festival, free tours of historical and cultural sites, and an educational programme focused on the ecological and cultural heritage of their town in collaboration with several schools. They co-operate with other local community organizations for example art galleries, museums, hiking group and a fishing co-operative. It is run and managed by its founder, and currently employ 2 staff members, 3 at the time of data collection. Additionally, it relies on many volunteers and informal labour and transactions for many of their activities.

CUA is both a workers’ cooperative and network of associations in rural Austria. Initially founded as an association in 2010 with the idea of free open space for experimentation, funded through the municipality. While there are several CUA associations, the study is focused on one location in a small village in Upper Austria, located in a technical school. The co-operative (CUA Co-op) emerged out of the association, in 2014, as the volunteer members of the CUA association began to take on larger projects that required lots of time, thus paid labour. The co-op is a workers co-operative, composed of individual member-owners and employees, with a focus on project-based work. In this particular case study, the lines between both sometimes are blurred, as some are members of both the associations and the co-op. The coop provides a lot of resources to the association, who rely on support from the municipality for its functioning, while it also receives donations from individuals and businesses. For clarity, ‘CUA’ will refer to the CUA association, and the CUA co-op will be stated when referring to the co-operative. The co-op, may be regarded as a form of social innovation, however, this study wants to understand SI processes through hubs, and for the sake of comparison it was better to focus on the association.

CUA offers firstly an open space for people (young and old) to meet, socialize, and share their ideas. Through this mix, they have been able ‘activate’ local people to share knowledge and be open to new ideas. This has also given rise to a number of new associations or businesses. CUA collaborates with many institutional partners, municipalities, businesses, schools, banks, local businesses.

***Description of the localities.*** The case studies are two small towns in Western Greece (population 14,386), and Upper Austria (7,602). Table 1 shows the stark economic contrast between the two regions, with Austria higher on economic indicators, with a GDP per capita in Upper Austria almost four times than that of the region of Western Greece.

**Table 1.** Regional Demographics of the two regions in comparison to the country average ( JRC, 2024).

Regional Demographics					
	Region of Western Greece (EL63)	Greece Average	Upper Austria (AT31)	Austria Average	EU 27 Average
**Population**	639, 500	10.4 million	1.53 million	9.1. million	NA
**GDP per Capita at current** **prices**	16, 520	23, 450	59, 580	57, 830	40, 810
**Employment rate %**	62.4	63.3	80.8	77.3	74.6
**% of persons at risk of** **poverty or social exclusion**	40.9	30	11.6 (2018)	17.5 (2020)	NA

While it would be easy to present and compare the classic economic based differences between the two countries, this study aims to explore the contextual environments and how this leads to different arrangements of SI, while also acknowledging the very different starting points across the world for enacting post-capitalist possibility. In Austria we have a strong market economy, with lots of state support for projects and regional development. In Greece, we see the opposite, with rural areas lacking in markets and state support, however informal activities play an important part in everyday life. Here we will present the two ‘local assemblages’, a complex assemblage of past and present, stories, institutions, culture, economies, knowledges and heritage that combine to create the local assemblage ( Willett, 2013)

*Western greece*

The regional demographics table shows that it has a GDP per capita almost 7,000€ lower than the rest of the country, a lower employment rate by 4% and is 10% higher regarding persons at risk of poverty or social exclusion ( JRC, 2024). This is a common feature of the Greek periphery, with most economic activity concentrated in Athens. More specifically, the town in question suffers from a narrative of decline, the effect of several events, shocks, policies among other factors. Literature on rural areas describes how these events collectively form a ‘vicious circle of decline’ that lead to their peripheralization ( Bock, 2016). Prior to 2018, the town and its economy were centred around a university with 5000 students, through hospitality services and student accommodation. However, as part of austerity measures, the national university system became more centralized, and most of the university’s departments were relocated to Patras, the closest city. Now only 500 students remain, representing a 90% decline. This had a significant impact on the economy, something that is clear if you take a walk through the main streets and observe all the ‘for rent’ and ‘for sale’ signs on former commercial buildings.

Despite the negativity and lack of opportunities, the area has an abundance of natural beauty. It is located on a large lagoon, which provides a natural habitat to several migratory birds and aquatic life such as eels and various fish. Fishing is part of the local culture, with fishermen living in a unique style of house on the water called *pelada*, with similarly unique fishing technique for the lagoon using netted pools and specialized netted boats. The town also is significant site for Greek national heritage, with it being one of the last sites of resistance against the Ottoman Empire in 1826. The siege is recognized as an important event towards gaining of Greek independence. It is celebrated yearly with a week of festivities and is heavily supported by the municipality. As its located on the open water, it has historically been open to many different types of people and cultures. People are very proud of their history, and of their town. There is a specific accent and words only used in the area, a book at the hub referred to this, with over 200 phrases particular to the local area. Tourism is seen as having big potential by many to save the towns economic fortunes; however it is clear some capacity is lacking to accommodate for them, there is a large hotel that is not fit for purpose, appearing derelict, mirroring what one interviewee described as one of many ‘almost places’ in the town.

*Upper Austria*

In Upper Austria, we have quite a different picture. The GDP is higher than the national average (€59,580 compared with €57, 830), employment is also higher (80.8% compared to 77.3%). Generally, the town appears to be doing ‘development’ right, the area is growing with new houses being built and lots of industry close by that provides plenty of jobs, and a relatively new motorway beside the village that links with nearby cities of Linz, Wels and Salzburg, and relatively well-connected public transport, given its peripherality. Located within the region of Upper Austria, it is part of one of the 2024 European Capitals of Culture, Salzkammergut. It is a very small village, of just over 7,000 inhabitants, yet functions as a commercial centre for surrounding towns and villages, with a couple of supermarkets, some restaurants, a bank, school, and a church. It is very clear that there is a lot of material wealth, it’s common to see swimming pools in people’s back gardens, nice cars, while electric bicycles can be seen cycling around.

However, there are social issues that affect the village, mainly an ageing population, and a lack of young people. One reason given for the decline of young people was the lack of services and activities that address the youth that does live there. Others noted the educational options, as the village has a technical school but not ‘Gymnasium’ second level school (more focused on academic education, sciences, languages etc.), which can cause children to leave at a very young age and lose ties with the village. Participants also spoke of a lack of public investment in public infrastructure, such as the school. Moreover, there are traditional and conservative values that some people can find constraining, such as views on gender roles, or politics (such as a conservative government in the region) thar progressive people saw as problematic.

Similarly to the Greek case, there is a specific Austrian dialect (more particular to the region than the place), although it does not have that specific local history and culture as the Greek case. It shares regional traditions, such as on Sundays it was common to see people dressed in traditional Austrian attire, men in ‘Lederhosen’ and women in ‘Dirndl’. The uniform for women working in the traditional Gasthaus’s is this traditional attire. People gather once a week in the Markthall for a market with local products. However, some interviewees explained that despite being well connected, local cultures and traditions are not shared between towns and villages in the region. Therefore, these narratives still mirror some of the classic depiction of rural areas, lacking in state investment, declining services and infrastructure supports, traditional values ( Edensor, 2006).

## Findings section

### CWS and local social needs

What is quite clear in Western Greece, is that many of its social problems are structural. The main problems that people stressed were rubbish (not being collected, public littering) and poor infrastructure – physical (e.g. roads) and social (e.g. places for exercise, playgrounds for kids). These material problems, they feed into the immaterial, regarding the feeling of inertia, narrative of decline, and people not taking responsibility about the town’s problems. This is what WGH aims to address, the founder stresses that “we cannot build roads, or deal with stray dogs so they don’t bite people” and that addressing these problems are the responsibility of the municipality to fix. This is shown through how WGH sometimes organize community clean ups of the lagoon, but don’t want to organize it regularly, as they would be doing the municipality’s job for them.

Rather than directly address the material problems of their town, the CWS addresses the immaterial element, as is clear in other SI initiatives ( Chen *et al.*, 2022). They aim to bring change rhizomatically, through everyday actions rather than the big events that are seen as providing economic development for the town by the municipality. While such events create a buzz, both in their duration and towards their build up, on normal days there is nothing happening, and the town feels empty.

MBL plugs itself into the local assemblage, one which was described above through having interesting history, culture, and traditions. They use the expressive and semiotic components of the local assemblage, and combine them with their vision of the town, and what it could be. For example, they offer engraving classes (a traditional practice from the area) where participants can engrave from photographs of the lagoon and the fisherman’s dwellings, or something else if they would like. They are very active on social media, always capturing a sunset or candid pictures of the lagoon.

They listen to their community to understand their needs. For example, the creative children’s activities exist because it was requested by parents. The creative activities vary weekly and can include making paper, creating notebooks, painting, animation workshops, screen printing to name a few. The founder explained that when he was younger, creative activities were scarce, there was only football and he had no idea you could be an artist, a photographer, a graphic designer etc. Along with being creative and learning new skills, the workshops are aimed to get the children working together. They try to pair older kids with younger, passing on the knowledge the older ones have learned in the classes. The hub also acts as a meeting point. While hosting different events and exhibitions, the different activities that take place more regularly act as an excuse for locals to meet and chat. It was very common for parents of kids to gather outside the hub on a Saturday with a coffee after they collected or dropped off their children. One day the founder described it as a cafe because of all the comings and goings. On some occasions people would stop by and discuss everyday matters, such as local politics, planning for the upcoming festival, or to give out about the available mezze options in the town. Thus, the CWS acts as an important and much needed meeting and focal point, and social infrastructure ( Klinenberg, 2018) for the town, along with providing creative activities that are also lacking.

It acts as a place where different people who would not normally cross paths can meet. Their activities address all ages, some engraving groups had participants ranging from 14 to 70. They have an oral history group that aims to retain oral traditions of the town, remembering specific people and events. They also collaborate with an ecologist and a local fisherman’s collective to provide an educational programme for children, working with several local schools.

These activities are provided to address the local needs of the community but are part of a bigger picture. They form part of a larger whole, an arrangement of desire, which, as shown before, is territorialised through the combination of different local components, such as local culture and natural resources. WHG has a vision of a future that it wants to produce. Their vision and desire for the town is one that is flourishing, where people take an active role in society, not “waiting for the great mayor” (Quote from interview with the manager) to come and fix things, but through small everyday actions they can construct a better future. Regarding their economic struggles, they see slow tourism as a solution. However, beyond the economic, they want a vibrant cultural life, people to care for and understand the environment, have local people engaged in activities outside their job and be involved in community activities. Through the existence of the CWS and its activities they offer a window into the future they desire to create – a community economy.

In CUA, we see a similar arrangement of desire. While they do not focus on local culture and traditions, they also make use of several material (e.g. technology) and expressive (e.g. open and collaborative ethos, ‘play’ mentality) to do so. While the local ‘economy’ is strong, they are lacking in some of the ‘social’ aspects of life. Despite local associations existing - music groups, a football club, photography, church groups etc, they were described by one CUA member as only having older people, closed and competitive. CUA is the opposite, promoting youth involvement, being open and collaborative.

Furthermore, the CWS has an alternative view on economy, and is more explicit regarding its political subjectivity, with the CUA co-op being a member-owned co-operative. Their impact report critiques the endless growth-and-innovation society we find ourselves in, and that CUA ‘ *challenges this through the creation of resonant connections between people, things and the world’* (CUA Impact report, 2020). The municipality is an essential component, without which the model of free open space would not function, as the municipality pays its bills and provides it with public infrastructure to use.

This subjectivity plays a key role in territorializing their assemblage, and broader societal change is much more present in their narratives rather than the focus on local development by WGH. However, they are not ideological, nor rigid politically and collaborate with actors they would otherwise criticize such as various municipal, state, and market actors (e.g. commercial banks). The social change they envision, is a collaborative over divisive, one where people are active in civil society, and not only focused on waged work, hence their suitability for transitions to post-capitalism ( Chatterton & Pusey, 2020). They have a focus on ‘new work’, inspired by the work of Frithjof Bergmann believing life should be split between paid work, community work, and leisure time to pursue one’s interests. The members of the co-op mainly live in this way, through self-employment, community work for the co-op through workshops or community activities in the association, while using their free time to ‘do what they really, really want’. Their activities are open to anyone in the community to join, and they have a full schedule of activities running that can be accessed online – art classes, technology classes for the elderly, repair café, woodwork, climate action group. Most of the focus is ‘being-in-common’, without competition, the need to succeed or achieve anything, as one interviewee highlighted.

They have a long history collaboration with the school, something that was addressed as a problem for the area – both the type of school it is (a technical school) and that it is old. Through their connections with industry, CUA co-operative provided over twenty 3D printers to the school, which are now integrated into the curriculum. They also have co-operated with certain teachers, doing different projects related to technology and independent thinking, and run similar projects funded by the regional authority with younger kids in preschool.

In both CWS, people form an essential component of their assemblage. In both locations, the CWS assemble interesting and unconventional people, that normally either are forgotten/left invisible in a small town, those who do not ‘fit in’ in the highly territorialized and codified (homogenous) small town. It seems the unconventional people can easier comprehend the change they wish to make, or they themselves desire change to a more open and inclusive society.

They both make sure they do not just employ anyone but want to ensure they have the same mindset and perspective, the same coding to ensure the territorialization of the CWS assemblage. In WHG, before hiring the staff, they were asked to write a personal essay on the town and the hub, why they think it is important. One of the staff members mentioned how he felt a magnetic attraction to the hub, as their goals are the same as his personal goals. In CUA, when looking to hire a new employee for the eGen, they put out an advertisement searching for someone “ *who is able to use a truck, able to work with wood material, who is open minded and able to have an open and mindful conversation with people.”* (Interviewee 1)

Moreover, both CWS have two leading figures that act as gatekeepers of the assemblage. Both may be considered as social entrepreneurs, and have a considerable impact on the assemblage, providing it with many affects. Such is common in SI initiatives, to have certain key individuals at the core of the assemblage ( Richter & Christmann, 2021).

### Changing social relations

In this section, we examine ‘changing social relations’, the second dimension of SI, considering the relational character of social relations through the concept of affect. Here we will explore how individual subjectivities transform into collective through the CWS. In both cases they do a lot of work to create community and increase collaboration. In Greece, the lack of collectivity in the periphery comes from a lack of trust in each other and institutions, often because of clientelist relations. In the Austrian periphery, while there exists strong institutional trust, there are a lack of places for socializing outside of the traditional meeting points or commercial venues. In both cases it was clear that many individuals lacked some form of a purpose in life, outside of their economic role. The CWS open space for diverse economic activities, on two separate, but related levels. Firstly, on the material level through praxis and secondly on the immaterial level in terms of people’s perceptions. Changing social relations and the subsequent increasing of affects and capacities towards community interests forms an essential part in the part of becoming post-capitalist subjects ( Gabriel & Sarmiento, 2020). Moreover, these actions have a dual character, as they also provide the CWS’ with affects and help with its everyday reproduction – an affective relation.

***Desiring Production and assisting with capacities.*** The CWS’s play a key role in desiring production, i.e. they help actors to create something (be it economic, social, or other). Both CWS’s act as nodes that give actors opportunities to affect and be affected, by providing them with resources, generally without charge, such as space, knowledge, materials, networks, equipment etc. that increases their capacities, and enables them to bring desires into reality, or in assemblage terms, desiring production. Part of their association comes from the ability of the CWS to provide meaning making, or axes of resonance ( Rosa, 2019) through non-alienated and significant relationships with the world and with others. Rosa (2019: 171) identifies resonance as a basic human capacity and need, and that all human desire, is a desire for resonance. It is this aspect that gives CUA’s motto – ‘CUA does not do anything, CUA makes possible’. This highlights how the CWS innovate not in an economic or technical sense but as *innovation in social relations*. They create and aid in the creation of new collaborations in places against the narrative that nothing is happening. We can regard this as SI practice, or outputs of SI processes ( Moulaert & Mehmood, 2020). It also highlights how the three dimensions of SI (addressing social needs, changing social relations and collective empowerment) are closely intertwined ( Moulaert & MacCallum, 2019). By addressing the needs of the local community (inertia in this case), they increase actors’ agency changing social relations and empower them with collective sense of identity.

One interesting example of this comes from an art teacher/artist, who first moved to Western Greece during COVID, working as a substitute art teacher. He initially found the CWS to meet local people, and once getting familiar with the CWS’ activities, collaborated with them for an engraving summer school, which evolved into workshop in the CWS’s annual festival, and later a workshop as part of their environmental educational programme with local schools. The teacher noted all these activities as being very important to him, improving his skills, and doing meaningful work. The hub also gained new capacities through the affective relation through gaining an art teacher to run weekly printmaking workshops and an artistic element to their educational program. The atmosphere of the CWS benefited, as the art teacher is a character and would call in frequently for small chats and gossip. An interesting observation is how this affective relation can affect the larger whole, the local community. From participating in the annual festival of the hub, it gave visibility to the art teacher, and he was approached by a local NGO that does artistic workshops with young kids and children with disabilities. In addition, through the educational programme, this affective relation offered an interesting solution to combat the lack of artistic infrastructure in the Greek public school system: Their solution entailed using the CWS as an art space, as part of their environmental education program –

“ *from my point of view, this is how the school has to be. An art classroom, which is ready, and when I want to do a lesson I will have my projector, my materials, the cabinet, every child will have his corner, his seat, a room filled with drawings or experiments that children have done before... here we have found, I am certain, we have found the solution to that problem.”*          (Teacher, interview)

There were several other examples of the CWS collaborating with local people to help realize their desires, such as giving sound equipment to owners of a local tavern on their re-opening night (a big night for the town), helping an elderly woman publish a children’s book and giving a young girl books, links and historical information about the town, in order for her to do a presentation to 150 people about a historical event, where the hub marketed the event, provided her with lights and sound equipment to make it happen. 

Regarding staff, it used to employ two artists; one of them left and the other could no longer be paid because of the end of a state funding programme. However, this affective relation was incredibly important for the CWS, for the town to retain two young artists, and for the artists themselves, who could have employment in their hometown, develop their careers as artists, and do meaningful work (e.g. with children, the environment). As one interviewee stated, “ *[artist 1] and [artist 2] are kids that probably the town would have nothing to do if the hub did not exist”.* The CWS and the town gained two artists, who pass on their knowledge to young kids. The mutual benefit was highlighted when one artist took part in a prominent Greek animation festival, and the organizers came to do an animation workshop with young kids. Moreover, despite them leaving, the CWS is still interested in their professional and personal development, and they still collaborate on projects when they have the time and funding. 

We see a similar picture in CUA, at the core of what they do is allowing people to follow their ideas and interests, helping people to find “ *what they really, really want”* to do. This happens through CUA’s model of free open space where people can encounter new activities and interesting people. Sometimes this happens to the extent that people can follow ideas into a funded project, generally from municipal, regional, or national funds. For example, For example, the CWS has a climate action group that started meeting informally to create a discussion about renewable energy in the town, they have since carried out several renewable energy projects in several public buildings, funded by the municipality. Another project between CUA, the school and the European Capital of Culture funds entailed schoolkids working with CUA in groups to realize their ideas (such as a film night with friends, hosted in CUA). On occasions an informal group forms into a more coherent body, for example a food co-operative was incubated there, also a company that makes 3D printers. On the individual level, CUA can unlock desires that otherwise would not have been possible living in a small town. One of their employees began his relationship with CUA as a teenager, taking part in a soldering workshop, He since has a degree in computer science and works on technology projects with the CUA eGen co-operative. He credits CUA for him following this career path.

Here we can see the CWS’s acting as a relational tool, creating new relationships between locals. They act as a middle ground, assembling different actors, with material (e.g. equipment) and immaterial (e.g. funding, knowledge) components they previously would not have access to, that enables them to realize their own projects, achieving their own desires. And almost all these happen in the framework of diverse and community economies, with affective labour as a core codification and resource.

***From the individual to the affective community.*** SI theory tells us how SI processes can lead to collective empowerment by changing social relations from individual to community needs ( Gibson-Graham & Roelvink, 2009). This section aims to unravel how this occurs in the CWS assemblages, as individuals encounter the CWS, they form part of the CWS’ affective community ( Zink, 2019). The CWS affective community is not the classic internal paid-membership form as is found in more market orientated CWS but expands outside their walls. The relationality ( Escobar, 2021) of the CWS describes their ability to keep creating connections with humans and non-humans, recognising radical interdependence based on care and respect.

For example, WGH, as noted before, organizes an annual festival, with a focus on local culture, heritage, and traditions they create an affective arrangement that forms a collective identity. Given their lack of resources to employ the number of workers needed to plan and manage the festival, they make use of the affective labour of the local community to help them. The festival activities, like much of the CWS’ everyday activities create positive affects for the town, not only for the CWS, with workshops about local culture and traditions, history, kayaking on the lagoon, hiking the surrounding mountains, art exhibitions, concert among many others. These actions create a bond between the locals and their place, and the variety of actors that inhabit it. The festival acts as a temporary arrangement of desire enacted, bringing together collective action and affective labour to create a window into what could be for the town, if everybody pooled together, highlighting what is possible in a diverse community economy.

In CUA, they also change social relations towards collective subjectivities through affective labour. The monthly repair café in CUA epitomizes this. The members of the group offer their labour, knowledge, and materials to the community for free, while they collaborate with other repair cafes nearby to share materials and ensure they don’t clash timetables. Their impact report states how these practices of repair *are intended to contribute to self-efficacy and self-empowerment, going beyond the relationship of consumption with objects*. Another interesting group was the ‘death café’ where people would meet up and talk about death, changing a typically individual and personal subject into a collective one –

*“something I loved was the death café, where people talked about death for hours.. Just about the topic death. What do you think about death? What do you think about and how do you experience? What experience do you have with death and the setup was sitting around drinking coffee, eating cake and discussing death. I like the setup and the chance to talk about topics where you don't have the chance to talk about it at home with, the people you love.”*
                                                                                                                                                                                                                                                                                                                                                                 (Interviewee 2)

This collaborative ethos is also apparent with the many collaborators each CWS has. By being open, and not focused on profit, their ethos is reciprocated. As one CUA member stated, “i *f you're generous to people, normally people are generous the other way around”.* In CUA, they often receive donations of equipment that they can either use for parts for repair, or in the organization such as laptops, coffee machines, and kitchen utensils. These practices can manifest change at the individual level, forming new collective subjectivities, as people want to contribute to the community economy. One interviewee, formerly the chairwoman of the association, noted how being part of CUA affected her own ways of thinking, how old buildings can be used to benefit the community:

*And the thing is that the CUA philosophy changed my work within the community or is influencing how I see things in the community, like having a lost place or an unused house that we can do there something without money, and what to do in social life.*
                                                                                                                                                                                                                                                                                                                                                                 (Interviewee 3)

In WGH, because of their relatively lower financial capacities, donations can have a higher impact. The organization was gifted a drone for videoing the lagoon, a telescope for birdwatching, a new phone for taking high quality photos, something which greatly increased their capacities and allows them to reach more people and become more professionalized.

More than just changing relations among their community, the CWS’s influence other larger assemblages with their open and collective way of doing things. We may understand how CWS’s affect other assemblages, through the concept of relations of exteriority, that each component part of the assemblage is autonomous, and can form part of other assemblages. Through collaboration with schools, and their existing relationships with the environmentalist and fishermen, they created a programme to address the lack of connection with locals and the natural environment, and the poor infrastructure of the public schools in Greece. Recognizing the exteriority of relations allows them to teach the schoolkids, inform them, and allow them to bring their knowledge into other assemblages, such as their families, and other groups they are part of, something Avdikos & Pettas (2021) refer to as diffusion of the CWS collaborative culture to broader circuits. 

WGH educate the public about the local environment and its importance for the town, that it’s not a distant far away thing or merely a resource to be exploited. They work with the fishing co-operative, who fish seasonally, but the rest of the year still must operate the ‘Divari’ (traditional fishing structure) to maintain the lagoon, keeping it clean and refreshing it with enough clean water. The ecologist explained how only the co-operative does this maintenance work, and there is an issue with private fishermen trying to win fishing rights, who only fish for profit rather than working with the lagoon and the environment. Through this education, WGH reframes the relationship between the ‘hegemonic trio’ of economy-society-environment ( Miller, 2019), not as separate spheres of life, but as interconnected and contingent on one another with a collection of human and non-human actants that co-exist and co-depend in the same geographical space.

In the case of CUA, they have a long-standing relationship with both the municipality and the local school, spanning over 10 years. Their relationship with the municipality has the agreement that the municipality will pay for the space and its maintenance, but will not interfere with it in any way, that it is free of party politics, and they should not expect any kind of output or results. The CWS focus is on the local community, and therefore, like WGH, the value it creates is not for the CWS, but for the town. Adhering to the exteriority of relations allows the CWS’ to penetrate other assemblages, and to ‘infiltrate’ other more strictly coded and highly territorialized assemblages, such as schools and municipalities, while maintaining their own autonomy and not adhering to their codes.

Through this symbiosis the school also benefits from their existence, through shared projects, a summer school and donation of 3D printers. The local community also benefits from the re-coding of property to the community through the opening of public property outside of school hours. For example, a wood turning group uses the woodwork room. While initially difficult to understand, one teacher explained how it changed their own perceptions about the school infrastructure:

*you know it's only for the short period when it's being used, then actually for the majority of the day it’s completely empty…. this was this was new for us, but some ones come in my arts room. It's not my arts room, but I thought about it. Why do I react in this way?*

The emancipatory of the potential of these practices lies in these two levels of changing social relations, desiring production (praxis) and changing individual to collective subjectivities. This is emancipatory and transformative as it destabilizes what Deleuze and Guattari describe as the machinic unconscious, unlocking a revolution of micropolitical becoming in those that encounter the CWS’s. Rather than being subjected to machinic enslavement and inertia, the CWS’s offer liberation from the circuits of the machines at play in both localities, be it through the ‘left-behindness’ in Greece or lack of purpose in Austria. The use of affective labour has transformative potential, as it creates collective subjectivities, social networks, community, and biopower from below ( Hardt, 1999).

## Discussion: the transformative potential of CWS

The third dimension of SI, according to Moulaert & MacCallum (2019) is ‘socio-political transformative potential’. We would like to understand the type of transformation the CWS’s enact is enabling ‘intentional economies’ in the words of Gibson-Graham (2008) or ‘real utopias’ as Wright emphasised ( 2010). Both represent open ended post-capitalist realities, by facilitating pluriversality ( Escobar, 2021) or economic diversity ( Gibson-Graham, 2008), working towards creating many different worlds, rather than the perceived dominant capitalist world, destabilizing its perceived hegemony. Rather than providing an exact post-capitalist strategy, the CWS provide a “ *multitude of possibilities of what could come after, as well as building daily competences to leverage social change.”* ( Chatterton, 2016: 405)”. Moving beyond certainty, we aim to provide one potential pathway of many possibilities towards creating such a future. To do so, we would like to infuse Wright’s thinking with that of DCE, understanding social transformation as post-capitalism, and Wright’s three types of transformation, as three stages of transformation.

As highlighted in the literature review section of the paper, Wright provides three pathways towards social transformation – *ruptural, interstitial and symbiotic.* Interstitial transformation may be the social transformation most closely linked to grassroots SI, referring to the ‘ *various kinds of processes that occur in the spaces and cracks within some dominant social structure of power’* (p. 229). Wright gives examples of interstitial transformations such as various forms of co-ops, workers councils, community based social economy organizations etc., which are also common examples of SI. Such initiatives offer viable alternatives to state and capitalist ways of organizing, however, are often critiqued as operating in isolation, whereby they do not offer a real viable alternative, but merely as heterotopias that operate within ‘niche’ or ‘protective spaces’ ( Smith & Raven, 2012). This is what we conceptualize as our first level of transformation, which we can clearly see as the two CWS’s work towards satisfying community needs and build community economies, through desiring production and building affective relations.

Both spaces act as heterotopias, or syntopias ( Vidaillet & Bousalham, 2020), that allow users to experience other ways of being in the world. While both hubs offer this, CUA is more explicit about its interstitial function of the space – “ *we have, kind of research Lab to try out things outside of the normal economic, a free space where it's not based on the normal ongoing system status and that was one of the basic ideas with CUA to create the spaces, to create ideas. Maybe for the future. Maybe it is quite small, but maybe change something if we need.”* (CUA co-founder, interview).

Regarding symbiotic transformations, Wright differentiates them from interstitial mainly due to their orientation towards the state, which they use as a mediating body to reduce capitalist power, with the main example given as class compromise, where workers and capitalist firms negotiate through the state over workers’ rights, pay etc. However, we would like to offer a different conceptualization of symbiotic transformation, as a second stage of interstitial transformation diffusion, as they influence other spatiotemporal arrangements. This may initially be in antagonism, and lead to their practices being copied/replicated by state or municipal organizations, or it may mean these institutions recognising and supporting interstitial transformations as important social economy actors. In both cases we also observe this level of transformation, with long term institutional collaboration of CUA and the municipality and more recently in WGH as they have begun to collaborate with the new municipality through organizing a public consultation regarding the town’s development. Moreover, both CWS also offer their resources and capacities to local actors such as schools, local businesses and other market entities, NGOs, and other local associations, blending DCE economies with the market economy. While interstitial transformation through the DCE framework is usually performed inside the CWS, symbiotic transformation happens outside the bounded environment of CWS and offers to other entities and the state to experience the alternative socio-economic arrangements, through the DCE framework that CWS’s assemblages perform.

Within SI literature, SI is often deemed to be transformative when it pierces this institutional level ( Haxeltine *et al.*, 2016; Moulaert & MacCallum, 2019), when SI bottom-up initiatives are supported by institutions, it is referred to as bottom-linked SI that *“develops when citizens' collective initiatives result in agreements with local institutions that enable and sustain such initiatives through sound, regulated and lasting practices”* ( Garcia *et al.*, 2015: 93). This symbiotic approach has many merits and is key for SI’s transformative potential to move beyond their space in the margins. However, SI being recognized by institutions does not equate to the neoliberal rhetoric by governments over the last twenty years (for example see Fougère *et al.*, 2017 for a full discussion), something that ‘limits social innovation to marginal change’ ( Bartels, 2017). Initiatives need to be given resources and power rather than just responsibility ( Bragaglia, 2021). Moreover, through symbiosis it allows us to move beyond the binary of initiatives either rejecting institutions or being co-opted by them, allowing for real change to be enacted and further institutionalized ( Bartels, 2017).

**Figure 1.**
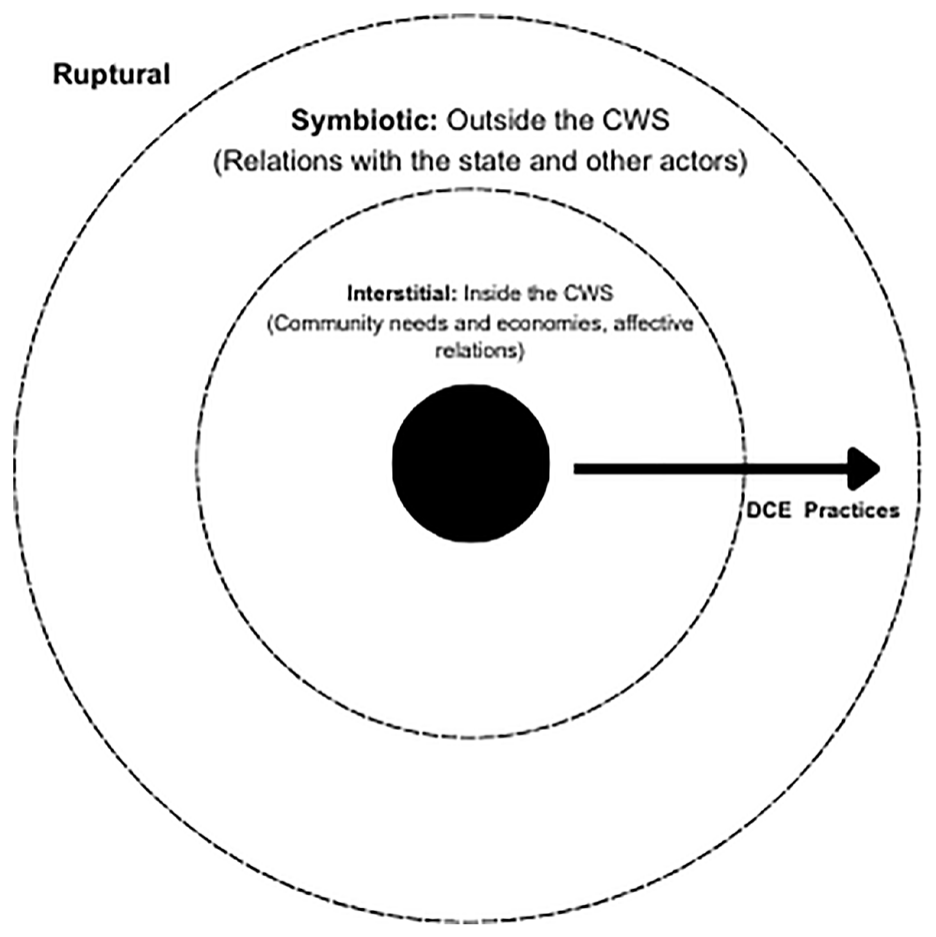
How Diverse and Community Economy practices diffuse throughout the three levels of transformation.

Figure 1 demonstrates how as DCE practices are performed and practiced, moving from the edifices and margins as interstitial transformations, to piercing the institutional dimension, and beyond we envisage CE practices spreading in a form of contagion until they reach a saturation point of post-capitalist becoming, signifying not necessarily a straight clean break from capitalism to post-capitalism, but a revolution of becoming, as different aspects of life become recoded and DCE are heavily territorialized and enacted in society. Both cases do not reflect this level of transformation (yet, at least), but an example of such a ‘real utopia’ would be the widely documented case of Mondragon in the Basque country, with the co-operative model completely institutionalized in the region. While as an example it is highly contextual and non-replicable, it gives an example of possibility ( Gibson-Graham, 2008). We cannot state this is the case for either case studies, both remain still in their symbiotic stage. The passage to a ruptural transformation requires CWS to be supported by various local and extra-local actors to be acknowledged as the only alternative and post-capitalist solution, where DCE can become the dominant mode of production and gain a hegemonic place in society.

## Conclusion

The paper discussed the role of CWS as initiators and facilitators of SI processes in two community led CWS in two rural areas in Austria and Greece. The research methodology employed a qualitative case study approach, utilizing participant observation and semi-structured interviews to explore the multiple sets of relations found in these rural CWS, aiming to understand how these arrangements cover local needs and foster new social relations. The findings reveal that CWS play a significant role in addressing social needs and facilitating grassroots SI practices, creating affective relations that contribute to the transformation of individual to collective subjectivities. CWS seem to provide a site where the unmet needs of the local people are negotiated, redefined and are covered in a co-creative and collective manner. This is happening in a DCE framework, where affective labour mostly performs.

CWS have a significant impact on individual subjectivities by transforming them into collective subjectivities through affective relations and desiring production, through the implementation of collaborative projects. The CWS play a crucial role in changing social relations and increasing affects and capacities towards community interests, which is an essential part of becoming post-capitalist subjects. They provide opportunities for actors to affect and be affected, increasing their agency and assisting them with their own desires by providing resources such as space, knowledge, materials, networks, and equipment. This process enables desiring production and creates non-alienated and significant relationships with the world and with others, which is essential for subject formation and the development of collective subjectivities.

Furthermore, the affective relations formed within CWS's have a transformative potential, as they create new collaborations and relationships in places where the narrative that nothing is happening. These affective relations also extend to the larger community in a symbiotic manner, creating positive affects for the town and fostering collective action and affective labor to create a window into what could be for the community. Additionally, the CWS's influence other larger assemblages through the concept of relations of exteriority, allowing them to 'infiltrate' other more strictly coded and highly territorialized assemblages, such as schools and municipalities, while maintaining their autonomy.

Future research could explore similar community led CWS assemblages in other contexts, including outside of Europe, to see what we can learn and how their transformative potential can be institutionalized. Furthermore, both cases were mainly led by an individual, who had a significant impact on the overall assemblage, regarding the resources they put into it and their decision making. Their power seems to have a (dis)empowering ( Avelino *et al.*, 2019) impact on the overall assemblage, as despite their energy and intensity increasing its affects, it could sometimes be overwhelming for other participants, causing one staff member in the Greek case to leave. Thus, future research could focus on more rhizomatic forms of SI initiatives, with less control of one individual, and how it differs to these SI assemblages.

## Ethics and consent

### Consent

Interview Participants were given an information sheet about the study and were required to sign a consent form prior to being interviewed. An ethical approval committee at Panteion University approved the study (Protocol Number: 44/ 30-9-2022). An example of the information sheet and consent form can be found above in the ‘extended data’ section.

Regarding participatory observation, the researcher made his identity as a researcher known to the users of the CWS by contacting the CWS in advance via email, requesting that they inform relevant stakeholders that there will be a researcher present for the given time period, and to notify them should they not consent to participate in the study to contact the researcher through contact details provided. When present on site, the researcher made his identity as a researcher known to the those participating in the CWS’ activities, and verbal consent was required because of the short time duration of their visits. Moreover, although minors took part in some workshops (as stated in section 5.1.), the participants in the workshops were not interviewed or subjects of participant observation, and therefore it did not require consent.

## Data Availability

Due to ethical restrictions on data sharing should any reader or reviewer request data to be made available, small sections of de-identified quotes may be available on request by emailing the corresponding author. ( colmstockdale@panteion.gr) Figshare: Colm_Stockdale_Information_Consent_Form. Stockdale, Colm (2024a). Colm_Stockdale_Information_Consent_Form.docx. figshare. Online resource. https://doi.org/10.6084/m9.figshare.26356546.v1 *This project contains the following underlying data:* The information sheet and consent form I used for my study to inform participants about the study and obtain their consent for participation. Data is available under the terms of the Creative Commons Attribution 4.0 International license (CC-BY 4.0) ( https://creativecommons.org/licenses/by/4.0/). Figshare: Table 1 Regional Demographics, Stockdale, Colm (2024b). Table 1 Regional Demographics. figshare. Dataset. https://doi.org/10.6084/m9.figshare.26356738.v1 *This project contains the following underlying data:* A table comparing regional demographics between the region of Western Greece and Upper Austria under indicators such as Population, GDP per Capita at current prices, Employment rate %, and % of persons at risk of poverty or social exclusion. Data is available under the terms of the Creative Commons Attribution 4.0 International license (CC-BY 4.0) ( https://creativecommons.org/licenses/by/4.0/). Figshare: Interview Protocol. Stockdale, Colm (2024c). Interview Protocol. figshare. Online resource. https://doi.org/10.6084/m9.figshare.26370316.v1 *This project contains the following underlying data:* The document contains the interview protocol for the study. This includes the research question and sample interview questions asked to interview participants. Data is available under the terms of the Creative Commons Attribution 4.0 International license (CC-BY 4.0) ( https://creativecommons.org/licenses/by/4.0/). Figshare: Research Protocol. Stockdale, Colm (2024d). Research Protocol. figshare. Online resource. https://doi.org/10.6084/m9.figshare.26370331.v1 *This project contains the following underlying data:* This document contains the research protocol used in the study. It contains the timeline for fieldwork and the Data collection procedures (data protection, identification of data sources). Data is available under the terms of the Creative Commons Attribution 4.0 International license (CC-BY 4.0) ( https://creativecommons.org/licenses/by/4.0/).
