# Transformative social innovation and rural collaborative workspaces: assembling community economies in Austria and Greece

**DOI:** 10.12688/openreseurope.18007.2

**Published:** 2025-01-20

**Authors:** Colm Stockdale, Vasilis Avdikos

**Affiliations:** 1 Department of Economic and Regional Development,, Panteion University of Social and Political Sciences, Athens, L. Syggrou 136, 16761, Greece

**Keywords:** Collaborative Workspaces, Social Innovation, Diverse Economies, Post Capitalism, Assemblage, Rural

## Abstract

**Background:**

Collaborative Workspaces are rapidly growing and evolving across the world. Traditionally understood as an urban phenomenon, most research understands them as either ‘entrepreneurial-led’, as profit-driven and commercial spaces such as business incubators and accelerators, or ‘community-led’ as being bottom-up, not-for-profit ventures aimed at catering for the needs of their community. Recent years however have seen their diffusion beyond large urban agglomerations to small towns and villages, with their functions assumed to be more community-orientated. At the same time, social innovation, or social innovation processes have been gaining prominence in academia, policy, and practice, as they address societal problems and hold potential for new forms of social relations. This paper attempts to provide a novel framework towards understanding the transformative potential of rural collaborative workspaces, as they engage in processes of social innovation, by drawing from diverse and community economies literature and assemblage thinking.

**Methods:**

The paper uses international case study comparison between rural Austria and Greece (One case from each country). Methods applied were: semi-structured interviews (N=28), participant observation and focus groups (2).

**Results:**

Community-led rural collaborative workspaces hold transformative potential from i) their ability to assist rural actors with their capacities and realizing their desires and ii) changing individual subjectivities towards collective. Through changing social relations in praxis and perceptions, we examine how social innovation processes through collaborative workspaces can be understood as a means of opening new economic subjectivities towards creating community economies as their transformative potential.

**Conclusions:**

Although rural collaborative workspaces hold potential for societal transformation, they require further institutionalization and support to move beyond the interstitial and symbiotic stages of transformation.

## 1. Introduction

In light of the many and seemingly increasing societal challenges facing society today, there is need for a radical transformation of society, something which has become a fact across alternative and mainstream agendas ( Apostolopoulou *et al.*, 2022). Many of these calls for change critique the social, political and economic status quo that are “ *based on increasingly severe encroachments in social and ecological systems and leading to the highly unequal destabilisation of communities and ecosystems”* ( Schmid, 2022: 61). Such is the extent of these encroachments that we are now at the conjuncture of multiple and inter-related crises– climate, social, economic, political, economic – what Homer-Dixon *et al.* (2021) label as the ‘polycrisis’. Within this context, the last decades have seen a variety of new forms of social movements, organizational and collective forms that re-arrange human and non-human actors around contemporary issues from social justice to climate change, that aim to construct alternatives in the here-and-now, improve their respective situations, or work towards changing institutional forms of inequality ( Roelvink, 2016). Roelvink describes these as social innovation, however they share characteristics with a plethora of different but inter-related concepts, such as alternatives to development, degrowth, post-capitalism, alternative economies, solidarity economy and more. What they have in common is a critique of the status quo and the desire to create new social relations based on social and ecological justice ( Apostolopoulou *et al.*, 2022; Escobar, 2015; Schmid, 2022). This paper seeks to add to this growing body of knowledge of transitions discourses that call for paradigmatic or civilizational transformation ( Escobar, 2021) by providing a novel theoretical and conceptual framework for thinking about social innovation (hereafter SI), and its transformative potential.

Like other sustainability transitions discourses, (such as green growth vs degrowth), SI remains contested between entrepreneurial and collective understandings ( Montgomery, 2016). Although part of its transformative potential is its ability to “empower people” and “drive societal change” ( BEPA, 2014), these connotations also enable the ‘instrumental’ use of SI by legitimizing neoliberal politics and further erosion of the welfare state ( Marques *et al.*, 2018; Swyngedouw, 2005). On the contrary, SI in its grassroots form has the potential for radical change ( Apostolopoulou *et al.*, 2022). According to Moulaert *et al.* (2017) SI is i) a way of understanding a wide range of activities and practices oriented to addressing and *providing solutions to specific social problems* or meeting human needs. Moreover, SI ii) involves *changing relations* through the adoption of new social practices, institutional arrangements and/or forms of participation. However, the effects of SI extend beyond the immediate meeting of needs. SI seems to improve long term opportunities for individuals and communities, through producing more efficient, effective and sustainable means for society to deal with its challenges, and that iii) can have a deeper *transformative impact* over communities and society. However, this transformative impact remains vague, particularly regarding how it can be considered transformative and how it may come about, which necessitates moving beyond the SI literature to develop new theoretical insights ( Marques *et al.*, 2018). This paper seeks to elaborate a novel theoretical framework by drawing on aspects of assemblage ( Deleuze & Guattari, 1980) and post-capitalist utopian thinking ( Gibson-Graham, 2008; Wright, 2010) to both describe and further theorize the transformative potential of SI processes.

This will be examined empirically through Collaborative Workspaces (hereafter CWS). CWS have emerged initially in urban areas since the mid-2000s as coworking spaces, creative hubs, hackerspaces, fab labs, etc host nowadays more than 5 million coworkers worldwide (ergonomictrends.com). Although primarily an urban phenomenon, CWS have also started to spread in medium and small cities in the last few years, away from urban agglomerations ( Avdikos & Merkel, 2020). CWS are mainly understood as commercial ventures offering a service by providing workspaces to remote workers, while also facilitating professional encounters and networking possibilities. Recent years, however, have shone a light on the political economy of CWS, and particularly their emancipatory potential (e.g. Vidaillet & Bousalham, 2020; Sandoval, 2016; Smith, 2020). Particularly, CWS in small cities and towns can play the role of a socio-cultural center where locals can engage through new participatory ways of being, through different kinds of projects that benefit their localities and that can eventually end up and be described as social innovation processes. Therefore, a second empirical contribution of this paper lies in unpacking the role of CWS as initiators and facilitators of SI processes in rural areas, through the example of two community led CWS in Western Greece and Upper Austria. Through the analysis of qualitative data, mainly through interviews and participant observation, we delve into the multiple sets of relations found in these two rural CWS and attempt to unpack the ways these arrangements of SI have potential for social transformation.

## 2. Literature review: Social innovation and collaborative workspaces

### 2.1. Social innovation

The use of SI over the last 30 years has increased almost exponentially ( Van der Have & Rubalcaba, 2016). Within academia the term’s meaning varies across fields such as innovation studies, management studies, sustainable and territorial development ( Howaldt *et al.*, 2014). This wide array of understandings is said to both be of help ( Moulaert & MacCallum, 2019) and hinderance ( Bock, 2012) to understanding the ‘fuzzy’ concept that is SI (ibid). While not providing an exhaustive literature review of SI, this section will serve to unravel SI, while positioning the paper within the literature and allowing for a reconceptualization of the concept through Diverse and Community Economies ( Gibson-Graham, 2008).

As a number of researchers have emphasized, SI seems a ‘contested’ concept ( Ayob *et al.*, 2016; Marques *et al.*, 2018). On the one hand ‘social’ innovation can be understood as social entrepreneurship whereby social entrepreneurs come up with innovative ideas to address society’s most pressing challenges, while the other approach is more understood through collective action and the social and solidarity economy. We want to highlight the capitalocentric nature ( Gibson-Graham, 2008) of the entrepreneurial understandings of SI, and aim to demonstrate that SI is also not reducible to one actor, but it operates as a SI assemblage.

Within the capitalocentric strand, SI is understood as a technocratic, entrepreneurial/market driven complement to the neoliberal state (e.g. Mulgan, 2006; Murray *et al.*, 2010). Much of the focus is on individual entrepreneurship and mainstream innovation approaches to ‘solving’ society’s most pressing problems. Here, SI is understood as ‘ *an idea of a need that isn’t being met, coupled with an idea of how it could be met’* ( Mulgan, 2006: 149) or more specifically “ *new ideas (products, services and models) that simultaneously meet social needs and create new social relationships or collaborations. In other words, they are innovations that are both good for society and enhance society’s capacity to act.”* ( Murray *et al.*, 2010: 3). Such understandings promote a capitalocentric worldview, whereby civil society is viewed as an untapped source of innovative capacity that can be harnessed towards creating new markets and economic value. Such understandings have also pierced policy documents, with SI seen allowing governments to ‘do more with less’ ( BEPA, 2014), increasing growth and competition while addressing society’s most pressing problems ( Fougère *et al.*, 2017).

Several scholars critique this capitalocentric discourse, generally as it proliferates both neoliberal governance and subject production ( Bragaglia, 2021; Fougère *et al.*, 2017; Jessop *et al.*, 2013; Swyngedouw, 2005). In these interpretations, SI is understood as quick-fix solutions to social problems through the creation of active economical and entrepreneurial subjects for a capitalist economy ( Arampatzi, 2022; Fougère *et al.*, 2017; Jessop *et al.*, 2013), while shifting social and environmental costs from the state to citizens ( Fougère *et al.*, 2017; Swyngedouw, 2005).

Moving to the more critical strand of the literature, developed over the last 30 years primarily by Frank Moulaert and colleagues (e.g. Moulaert *et al.*, 2010; Moulaert *et al.*, 2013), who developed SI as a concept vis-à-vis neoliberal practices and policies, originally understood as an alternative model of local development ( Moulaert *et al.*, 2005). With its roots in new social movements ( Godin, 2012; Martinelli, 2010), SI is understood not as an organizational form, but as ethical spatial projects, by changing social relations towards meeting human needs and not as a gap in the market to be addressed. Moulaert & Mehmood (2020: 17), portray how many SI’s are rooted in socio-ecological movements, as consequences of increasing privatization, climate crisis and increasing social inequalities. Examples include the new-municipalist movements (e.g. in Madrid and Barcelona), the creation of new urban and rural commons, agroecological initiatives, local development experiments aiming to move past the market-based economy etc.

Thus, SI is broadly understood as *innovation in social relations by meeting human needs* ( Moulaert *et al.*, 2013). SI is understood as both a practice (collective satisfaction of human needs) and a process (changes in social relations, collective empowerment) ( Moulaert & Mehmood, 2020). Part of our contribution is through understanding the underlying processes that lead to collective action, SI can be understood as a means of opening new economic subjectivities and post-capitalist possibilities ( Gibson-Graham, 2008; Wright, 2010) which we would like to conceptualize as the transformative potential of SI, building through Assemblage thinking, as the analytical and methodological tool.

### 2.2. Collaborative Workspaces

CWS refers to spaces, which are primarily work purposed environments, but they can also be places where people come together for both work and other personal activities in a shared space such as coworking spaces, makerspaces, fablabs, business incubators and accelerators, social and cultural centres etc. ( Schmidt & Brinks, 2017). CWS have been described as sites of ambivalence, demonstrating tensions between both corporate and counter-corporate, capitalistic and commoning, collaborative and individualistic ways of being ( Bandinelli & Arvidsson, 2013; De Peuter *et al.*, 2017; Vidaillet & Bousalham, 2020). For example, the term may relate to work collectives, co-operatives for freelancers and other forms in response to precarious markets, which operate with values of the social and solidarity economy; or alternatively CWS may act as business incubators or accelerators for start-ups, along with corporate chains of coworking ( Arvidsson, 2020; Brooke *et al.*, 2014; De Peuter *et al.*, 2017). Contributions generally differentiate between entrepreneurial-led vs community-led CWS ( Avdikos & Iliopoulou, 2019; Avdikos & Merkel, 2020), with the latter also referred to as resilient CWS ( Gandini & Cossu, 2021). While both types overlap in their activities and functions ( Avdikos & Pettas, 2021), community led CWS generally are not-for-profit and have an authentic concern for their community ( Avdikos & Iliopoulou, 2019).

Such tensions between collectivity and entrepreneurship also reflect how CWS engage with SI. For example, more entrepreneurial and individualistic understandings are demonstrated by the franchise Impact Hub, that acts as an incubator for ‘changemakers’ such as social entrepreneurs and socially orientated organizations. On the other hand, there is a growing number of contributions referring to the political economy of CWS, and their potential for fostering alternative economic practices. For example, Sandoval (2016) demonstrates how co-operative CWS that ease precarity for freelancers, may simultaneously replace competitive and individualized work cultures with more collaborative ones. Other CWS, such as makerspaces engage can facilitate grassroots innovation practices when used by local citizens to address local social needs. For example, providing marginalized groups such as refugees in Greece or favela dwellers in Brazil with collective space for repair and community activities ( Dias & Smith, 2018; Liodaki, 2024). Moreover, socio-cultural centres have potential to engage with SI processes by offering their space to be used by the local community (e.g. neighbourhood associations) for events and other services ( Brown, 2017; Garcia *et al.* 2015; Stockdale & Marmo, 2023). For example, Garcia *et al.* describe two initiatives in popular neighbourhoods in Barcelona that are partly publicly funded and engage in a variety of cultural activities while also providing social services to the neighbourhoods such as a library and welfare services. Finally, more radical examples of SI include autonomous social centres and occupations that aim to offer a complete alternative to the state or market, while still offering various services and cultural activities for people to take part in ( Membretti, 2007 cited in Marques *et al.*, 2018). While not always offering a complete alternative to the market, it appears that a number of community-led CWS may oscillate between the market and the commons ( Avdikos & Pettas, 2021). These CWS thus can be used as a tool to foster economic diversity ( Gibson-Graham, 2008; Smith, 2020) by engaging in simultaneous capitalist, diverse and non-capitalist practices, acting as ‘syntopias’ ( Vidaillet & Bousalham, 2020).

More recently, the CWS phenomena has diffused to rural and peripheral areas, yet questions remain about how their dynamics may both compare and contrast to their urban counterparts. Recent research with data collected from 23 European countries (12,006 CWS in total) suggests that 66% of CWS are located in urban regions, while the rest (34%) are in intermediate or rural regions ( Marmo & Avdikos, 2024) ^1^. Multiple sources (e.g. Görmar, 2021; Tomaz *et al.*, 2022) have highlighted that CWS in rural areas are more diverse in the activities they offer than urban CWS, ranging from community services to social and cultural initiatives. Through offering a range of services, such CWS can act as a ‘social hub’, or ‘social infrastructure of care’ for communities, by both creating and maintaining the social fabric of rural areas ( Avdikos & Merkel, 2020; Merkel, 2023; Marmo & Avdikos, 2024). This paper focuses on two social-cultural centres in rural Greece and Austria respectively, and aims to answer the following research questions:

1) How do rural collaborative workspaces engage in social innovation processes?
2) To what extent can these social innovation processes have transformative potential for society?
3) How can diverse and community economies and assemblage thinking inform transformative social innovation theory?

## 3. Conceptual framework: Transformation of social relations

There is a growing body of research linking SI and societal transformation ( Moulaert & MacCallum, 2019). This growth can largely be attributed to the ‘polycrisis’ outlined in the introduction, in which various reputable international bodies have called for the need for society to change, such as the Intergovernmental Panel on Climate Change (IPCC) Working Group II and the UN calling for timely and significant society-wide transformational change ( Blythe *et al.*, 2018) What is more, rather than political will driving the need for transformation, it is out of structural necessity ( Rosa, 2019).

Regarding transformative SI, an important contribution to its theory has been that of the EC funded TRANSIT project to develop a theory on transformative social innovation (e.g. Avelino *et al.*, 2019; Haxeltine *et al.*, 2016; Pel *et al.*, 2020). Here, transformative SI is understood as a “ *process, through which social innovations challenge, alter and/or replace established (and/or dominant) institutions in the social-material context*” ( Haxeltine *et al.*, 2016: 19). The theoretical framework of the project comes from sustainability transitions thinking and conceptualizes transformative SI through the Multi-Level Perspective framework (MLP) ( Geels, 2004), whereby SI’s need to adapt to dominant rule sets in society and adapt to institutional logics (such as state regulations or collaborating with institutions) to become institutionalized and have a transformative impact in society ( Pel *et al.*, 2020). Despite the value however, there are several conceptual limitations on this approach, that are generally existent in most transitions’ literature. Firstly, there is a lack of critique regarding capitalism ( Chatterton, 2016; Törnberg, 2021) and secondly the MLP privileges structure and hierarchy, reproducing the very power relations responsible for the need for social change to take place ( Gillard *et al.*, 2016). In contrast, coming from an anti-essentialist and relational ontology inspired by Deleuze and Guattari’s Assemblage thinking and post-capitalist concepts, we aim to overcome such limitations.

### 3.1. Post-capitalist utopian thinking

Rather than adapting to the dominant structures in society, we follow Diverse and Community Economies (DCE) thinking with the idea that *“imagining and enacting radically different, sustainable, non-anthropocentric, postcapitalist futures is what we have here at hand.”* ( Gibson-Graham & Dombroski, 2020: 2). Moreover, Wright (2010) refers to alternatives that exist in the here and now, within and despite capitalist processes of enclosure, as interstitial transformations. Both Wright and Gibson-Graham offer examples of what Wright (2010: 4) terms ‘real utopias’ – *“proposals for pragmatically improving our institutions. Instead of indulging in utopian dreams we must accommodate to practical realities”.* DCE offers such practical realities through a discursive shift of the economy as a diverse space of ethical and political action. Rather than a dominant capitalist economy, Gibson-Graham (2006) ask us to read for difference over dominance, and understand the diversity of economic activity at play, such as *“non-market gifts, volunteer work, commons, cooperative forms of production, criminal economies and much more”* ( Smith, 2020: 596). Whereby, the ‘capitalist’ economy of wage labour, market exchange of commodities and capitalist enterprise becomes *“just one particular set of economic relations situated in a vast sea of economic activity”* ( Gibson-Graham, 2006: 70).

This non-essentialized understanding of economy allows us to see possibility even in perceived dominant institutions in society, by understanding the multiple transactions, labour arrangements, enterprise, property relations and modes of finance at play when performing economy ( Gibson-Graham & Dombroski, 2020). While diverse economies is a theoretical tool to understand the alternative production practices, the community economy refers to how these five areas of economy may be used to construct ethical economies ( Cameron, 2022). More specifically:

*“Community economy names the ongoing process of negotiating our interdependence. It is the explicit, democratic co-creation of the diverse ways in which we collectively make our livings, receive our livings from others, and provide for others in turn.”*
                        ( Gibson-Graham *et al.*, 2020).

Gibson-Graham & Roelvink (2009) use the example of Argentina post-2008 financial crisis, to demonstrate how SI processes can encourage the cultivation of community economies, with interstitial transformations emerging from the cracks of a crisis stemming from the capitalist economy – “ *the unemployed started to build community economies by engaging in barter and using alternative currencies, providing neighbourhood-based social services and schooling, and taking over factories and running them cooperatively”* (p. 32). What is key to these community economies was the processes that preceded them – “ *To transform themselves into community economic subjects, they created a cooperative radio station; they went to the World Social Forum in Porto Alegre to see themselves reflected in others who were also engaged in projects of self-determination.”* (p. 32).

Therefore, rather than thinking about transformation in the sense of radical transformation through overthrowing a monolithic system, and replacing it with a better system, or a meta-narrative of how the future will unfold, DCE and other post-capitalist thinking deals with the here-and-now, and embraces the multiplicity of non-capitalist and more-than-capitalist practices that already exist, the possibilities that could unfold as a result, and building competencies to leverage future social change ( Chatterton, 2016; Miller, 2019). To help conceptualize some steps in the process of transformation, we will draw from Wright, who identifies three main ‘pathways’ for social transformation: radical ‘ruptural’ transformation through wide-scale revolt against state and market, while bringing in radical alternatives; ‘symbiotic’ transformation is through long term co-operation with existing structures and institutions (e.g. trade unions); while ‘interstitial’ transformations take a prefigurative approach, by creating future alternatives in the here and now. In the discussion section, we will further elaborate on this schema for transformation, moving from interstitial to symbiotic to ruptural, while stressing it is more of a roadmap rather than an essentialized vision of how a post-capitalist transformation will unfold. Thus, transformation is not a straight arrow or a specific end product, but a constant open process of struggle and deliberation ( Gibson-Graham & Roelvink, 2009).

### 3.2. Assemblage thinking

To give these re-conceptualizations of SI further analytical power, we draw on Assemblage thinking ( DeLanda, 2006; Deleuze & Guattari, 1980) as both an analytical framework, and ontology for transformation. The main contribution of assemblage is analysing socio-spatial phenomena and understanding them relationally as dynamic wholes, in constant flux, or becoming. Rather than their essential qualities, it gives priority to relations and qualities, enabling a mode of analysis where we can compare bodies through their given affective relations ( Buchanan, 1997).

The term ‘assemblage’ is a translation from the French - *agencement* - originally applied by Deleuze and Guattari to signify the capacity to act. Agencement holds more meaning than the English ‘assemblage’, including to arrange, to dispose to fit up, to combine, to order ( Law, 2004: 41). Therefore, while assemblages are made up of material, expressive and symbolic components, we must look at them beyond a collection of things but their interaction, arrangement, ordering and (dis)assembly. Moreover, we must look beyond the components themselves, which are just the “props needed to actualize a particular arrangement of desire.” ( Buchanan, 1997: 65), thus assemblages are desire driven. Moreover, central to assemblage thinking is the concept of affect, understood as the capacity to affect and be affected. When bodies enter affective relations with one another, it refers to those ‘encounters’ that may augment or diminish their respective capacities ( Slaby & Mühlhoff, 2019). This is central to our assemblage analysis, as we will demonstrate how various (human and non-human, material and expressive) component parts affect the assemblage and its capacities. This autonomy of parts can also be referred to as the exteriority of relations ( DeLanda, 2006). Affective labour ( Hardt, 1999) understood as the production and manipulation of affects, will also be essential to our understanding of the CWS role in organizing social relations, and subject formation

Beyond its analytical merit however, it is important to note at the core of Deleuze & Guattari’s thinking was their use of assemblages as “ *tools to be used to take apart and reconstruct the present order of things* ( Russell *et al.*, 2011: 582). Thus, assemblage thinking along with being an analytical tool, it also compliments the conceptual framework of DCE and utopian thinking. For example, DCE practices can be traced through relations within CWS assemblages to reveal their transformative character, such as a 3D printer being acquired from capitalist circuits but used as a common pool resource, i.e. introduced to commons circuits ( Avdikos & Pettas, 2021). Moreover, Avdikos & Pettas have conceptualized CWS as CWS assemblages, dissolving the internal and external physical boundaries of CWS, and understanding them as being composed of material and immaterial components. CWS assemblages also have a degree of ‘porosity’, that is the degree to which they are open or closed to an external community and engage in exchange of ideas and knowledge (ibid.). Following other approaches drawing from Deleuze & Guattari in trying to understand SI, we want to highlight the rhizomatic nature of social relations ( Pel *et al.*, 2020; Scott-Cato & Hillier, 2010). Thus, AT can complement DCE to reconceptualize SI processes through CWS, whereby changing social relations occurs as a non-linear, rhizomatic process that occurs through the culmination of everyday actions, without a necessary beginning or end. Through assemblage thinking, transformation is rhizomatic change through a micropolitics of becoming, seeking to open new possibilities and multiplicities, towards post-capitalist futures.

## 4. Methods

### 4.1. Research design and methodology

The research applied a qualitative case study approach ( Yin, 2018). Data collection was carried out for roughly one month in each case between March to June 2023. The study applied qualitative research methods: i) participatory observation, ii) semi-structured interviews, and iii) focus groups. A key informant (the main founder of the CWS) was used to gain access to the CWS, local people and various activities and events that took place during the stay. Prior to fieldwork, a research proposal was submitted and approved by an ethics committee at Panteion University (Protocol Number: 44/ 30-9-2022). Ethnographic methods are common for studies engaging with assemblage thinking, as they open the researcher up to ‘everyday and mundane’ practices, allowing for the recognition of non-human agency, their interaction with humans, and resultant effect on capacities ( Ghoddousi & Page, 2020: 6). The researcher engaged in over 300 hours of participant observation in both areas, with the role changing throughout the data collection period, from a ‘participating observer’, ‘partially participating observer’ and ‘minimally participating observer ( Bryman, 2012), depending on the situation. Observations were supported by 30 semi-structured interviews (27 participants, with 3 interviewees interviewed twice). Interview participants were identified firstly through participant observation, secondly some participants were identified from the gatekeeper of each case, and finally others were identified through snowball sampling. Participants have pseudonyms in line with GDPR and ethics guidelines, and their descriptions can be found in Table 1 below.

**Table 1.** List of interview participants.

Site	Pseudonym	Role	Description
Western Greece	WG1	CWS Member	Leader
	WG2	CWS Member	Co-founder
	WG 3	CWS Member	Worker, Secretary
	WG 4	CWS Member	Worker, Employed Artist
	WG 5	Collaborator	Art Teacher
	WG 6	Collaborator	Ecologist
	WG 7	Collaborator	Fisherman
	WG 8	Collaborator	Owner of local Museum
	WG 9	Collaborator	Principal of School
	WG 10	Collaborator	Dance teacher
	WG 11	Collaborator	Schoolteacher
	WG 12	Collaborator	Tour Guide
	WG 13	Local	Daughter does art classes, often stops by to speak with owner
	WG 14	Local	Takes part in creative activities
	WG 15	Local	Studies in another city, comes back in summer and takes part in CWS' activities
	WG 16	Tourist	Photographer, participated in some of CWS' activities
	WG 17	Local Student	Took part in clean up organized by WGH. Not local
Upper Austria	UA 1	Co-op Member	Leader/Co-founder
	UA 2	Co-op Member	Co-founder
	UA 3	Co-op Member	Graphic designer
	UA 4	Co-op Worker	Software Engineer (eGen), Repair Café member (Association)
	UA 5	Co-op Worker	Worker - handyman
	UA 6	Association Member	Art Classes, Administration
	UA 7	Former Member	Former co-founder (eGen and Association)
	UA 8	Association Member	Member of Climate group
	UA 9	Collaborator	School Art teacher
	UA 10	Collaborator	Local Politician, participated in a collection organized by CUA for Ukraine
Total	27		

The interviews were transcribed and analysed thematically using MaxQDA whereby patterns within qualitative data sets are identified and subsequently analysed ( Braun & Clarke, 2006). Due to language restrictions, not every desired participant (e.g. representative from the municipality) could be interviewed, which represents a limitation of the study. The interview content varied depending on the role of the participant. The themes of the semi-structured interviews (refer to extended data section for more information) mainly consisted of the person’s role and relationship with the CWS and their perceptions of their local area, while interviews with the leader of the CWS were more in depth about the structure and working of the CWS. Moreover, one focus group was carried out in each location with both the founder and their partner. These were conducted at the beginning of the research to gain an overview of the CWS assemblage. The participatory observations enhanced these results, giving the researcher further insight into everyday routines, events, atmospheres and happenings around the CWS assemblages. Together, the methods applied gave the researchers insights the multiple components of each assemblage, and their relations that they are composed of.

### 4.2. Description of the cases

This paper presents two contrasting geographical contexts of Austria and Greece, two countries which epitomize the stark economic contrasts that exist in the north-south European divide. More than simply economically rich-poor divisions, Northern Europe is historically industrialized and urbanized with welfare state provision ( Leontidou, 2010) while Southern Europe has a *’special case of capitalism’, characterised by late industrialisation, large agricultural and tourism sectors, speculative urban development and family-based informal economy.”* ( Gkartzios, 2013: 161, citing King, 2000). The two different cases of capitalisms in North and South Europe, coupled with interesting case studies (one municipal supported and one independent) were key reasons for choosing the locations, and to prove for interesting comparison.

Austria is one of the wealthier countries in the EU, as demonstrated below in Table 2. It appears to have a supportive institutional environment for SI initiatives, through a ‘hybrid’ economic model with influences from both market-centred liberal Western and state-centred Eastern European models of governance ( Novy *et al.*, 2009). Consequently, the state has a history of supporting creative and socially innovative bottom-up initiatives since the 1980s. Greece, in contrast, is one of the poorer members of the EU and is still recovering from the 2008 economic crisis, of which it had the harshest recession in Europe, experiencing large rates of out migration, regional poverty rates, a decrease in household incomes and increased unemployment ( OECD, 2020b). Moreover, it has high regional inequalities, with one in two people living in either Athens or Thessaloniki (ibid.). Greece does not have the same economically supportive environment for bottom-up initiatives and is the most centralized country in the EU ( OECD, 2016). Culturally however, it has a long history of collective action, social movements and social and solidarity economy, because of the lack of state or market structures to address needs typically addressed by the welfare state in Northern Europe ( Malamidis, 2020). Thus, despite two contrasting socio-economic contexts, both still have enabling environments for SI to emerge, be it through the state-supported model of Austria, or grassroots organizing of Greece. Thus, the cases demonstrate how SI emerges not exclusively from bottom-up or top down, but that " *Social innovation presupposes a critical attitude towards existing systems and their inherent failures, as well as a search for social justice and the public good*." ( Bock, 2012: 62).

**Table 2.** Regional Demographics of the two regions in comparison to the country average ( JRC, 2024).

Regional Demographics					
	Region of Western Greece (EL63)	Greece Average	Upper Austria (AT31)	Austria Average	EU 27 Average
**Population**	639, 500	10.4 million	1.53 million	9.1. million	NA
**GDP per Capita at current prices**	16, 520	23, 450	59, 580	57, 830	40, 810
**Employment rate %**	62.4	63.3	80.8	77.3	74.6
**% of persons at risk of poverty or** **social exclusion**	40.9	30	11.6 (2018)	17.5 (2020)	NA

Moving on from the national contexts, the case studies are two small towns in Western Greece (population 14,386), and Upper Austria (7,602). For reasons of anonymity, just the regions will be referred to. The Table 2 above shows the stark economic contrast between the two regions, with Austria higher on economic indicators, with a GDP per capita in Upper Austria almost four times than that of the region of Western Greece.

Despite being strong across the economic indicators, we want to look beyond GDP and economic indicators and understand the qualitative as well as the quantitative data. For example, although Austria has one of the lowest disparities in GDP between regions per capita in EU, it has large regional disparities in terms of well-being dimensions such as jobs, safety and community, and has an ageing population ( OECD, 2020a). Moreover, recent voting trends have been far-right in European and national elections, something that Harvey (2020) contends is a symptom of an alienated population.

***Western Greece***

The regional demographics show that the town in the region of Western Greece has a GDP per capita almost 7,000€ lower than the rest of the country, a lower employment rate by 4% and is 10% higher regarding persons at risk of poverty or social exclusion ( JRC, 2024). More specifically, the town in question suffers from a ‘vicious circle of decline’ through a culmination of different factors ( Bock, 2016). Prior to 2018, the town’s economy was centered around a university with 5000 students, through hospitality services and student accommodation. However, as part of austerity measures, most of the university’s departments were relocated to Patras, with now only 500 students remaining. This had a significant impact on the economy, something that is clear from all the ‘for rent’ and ‘for sale’ signs on former commercial buildings in the town’s centre. In informal conversations with local people during the fieldwork, many stressed how dire the economic circumstances are along with expressing a mistrust of the authorities because of their inaction to change anything.

Despite the negativity and lack of opportunities, the area has an abundance of natural beauty. It is located on a large lagoon, which provides a natural habitat to several migratory birds and aquatic life such as eels and various fish. Fishing is part of the local culture, with fishermen living in a unique style of house on the water called *pelada*, with similarly unique fishing technique for the lagoon using netted pools and specialized netted boats. The town also is significant site for Greek national heritage, with it being one of the last sites of resistance against the Ottoman Empire in 1826. The siege is recognized as an important event towards gaining of Greek independence. It is celebrated yearly with a week of festivities and is heavily supported by the municipality. As its located on the open water, it has historically been open to many different types of people and cultures. People are very proud of their history, and of their town. There is a specific accent and words only used in the area, a book at the hub referred to this, with over 200 phrases particular to the local area. Tourism is seen as having big potential by many to save the towns economic fortunes; however it is clear some capacity is lacking to accommodate for them, there is a large hotel that is not fit for purpose, appearing derelict, mirroring what one interviewee described as one of many ‘almost places’ in the town.

***Western Greece Hub***

Western Greece Hub (WGH) is a non-profit organization founded as a legal entity in 2019. The organization emerged from a former local activist group concerned about the downward trajectory of the town. Their purpose is to strengthen the collectivity in the local community through the participatory mapping of the city and its culture, preserving its environmental wealth and the creation of programs of alternative cultural tourism and ecotourism. Since 2019 the organization has a ‘Local Hub’ which acts as a node for community activities of the town. Among their many activities include a gift shop with local products, boat tours, art classes, creative activities for children, providing a meeting point for groups, Info Point, an annual cultural festival, free tours of historical and cultural sites, and an educational programme focused on the ecological and cultural heritage of their town in collaboration with several schools. They co-operate with other local community organizations for example art galleries, museums, hiking group and a fishing co-operative. It is run and managed by its founder, and currently employ 2 staff members, 3 at the time of data collection. Additionally, it relies on many volunteers and informal labour and transactions for many of their activities.

***Upper Austria***

In Upper Austria, we have quite a different picture. The GDP is higher than the national average (€59,580 compared with €57,830), employment is also higher (80.8% compared to 77.3%). Generally, the area is growing, with new houses being built, industry close by that provides jobs, a relatively new motorway linking with nearby cities of Linz, Wels and Salzburg, and a relatively well-connected public transport, given its peripherality. It is a very small village, of just over 7,000 inhabitants, yet functions as a commercial centre for surrounding towns and villages, with a couple of supermarkets, some restaurants, a bank, school, and a church. It is very clear that there is a lot of material wealth, it’s common to see swimming pools in people’s back gardens, nice cars, while electric bicycles can be seen cycling around.

However, there are social issues that affect the village, mainly an ageing population, and a lack of young people. One reason given for the decline of young people was the lack of services and activities that address the youth that does live there. Others noted the educational options, as the village has a technical school but not ‘Gymnasium’ second level school (more focused on academic education, sciences, languages etc.), which can cause children to leave at a very young age and lose ties with the village. Participants also spoke of a lack of public investment in public infrastructure, such as the school. Moreover, there are traditional and conservative values that some people can find constraining, such as views on gender roles, or politics (such as a conservative government in the region) thar progressive people saw as problematic.

Similarly to the Greek case, there is a specific Austrian dialect (more particular to the region than the place), although it does not have that specific local history and culture as the Greek case. It shares regional traditions, such as on Sundays it was common to see people dressed in traditional Austrian attire, men in ‘Lederhosen’ and women in ‘Dirndl’. This is also the uniform for women working in the local taverns However, some interviewees explained that despite being well connected, local cultures and traditions are not shared between towns and villages in the region. Therefore, these narratives still mirror some of the classic depiction of rural areas, lacking in state investment, declining services and infrastructure supports, traditional values ( Edensor, 2006).

***Collaborate Upper Austria***

CUA is both a workers’ cooperative and network of associations in rural Austria. Initially founded as an association in 2010 with the idea of free open space for experimentation, funded through the municipality. They have a focus on ‘new work’, inspired by the work of Frithjof Bergmann believing life should be split between paid work, community work, and leisure time to pursue one’s interests. The members of the co-op mainly live in this way, through self-employment, community work for the co-op through workshops or community activities in the association, while using their free time to ‘do what they really, really want’. While there are several CUA associations, the study is focused on one location in a small village in Upper Austria, located in the technical school. The co-operative (CUA Co-op) emerged out of the association, in 2014, as the volunteer members of the CUA association began to take on larger projects that required lots of time, thus paid labour. The co-op is a worker’s co-operative, composed of individual member-owners and employees, with a focus on project-based work. In this case study, the lines between both sometimes are blurred, as some are members of both the associations and the co-op. The coop provides a lot of resources to the association, who rely on support from the municipality for its functioning, while it also receives donations from individuals and businesses. While the co-op may be regarded as a form of social innovation, this study wants to understand SI processes through CWS, and for the sake of comparison it was better to focus on the association.

## 5. Findings section

### 5.1. Collaborative workspaces and local social needs

Many of the social problems in Western Greece are structural. The main problems that people stressed were rubbish (not being collected, public littering) and poor infrastructure – physical (e.g. roads) and social (e.g. places for exercise, playgrounds for kids). These material problems feed into the immaterial - inertia, narrative of decline, and a lack of responsibility regarding town’s problems. This is what WGH aims to address, the founder stresses that *“we cannot build roads, or deal with stray dogs so they don’t bite people”* and that addressing these problems are the responsibility of the municipality to fix. For example, WGH sometimes organize community clean ups of the lagoon, but do not organize regularly, as they would be doing the municipality’s job for them. Instead, the CWS addresses the immaterial, like many SI initiatives ( Chen *et al.*, 2022). They aim to bring change rhizomatically, through everyday actions rather than the big events that are seen as providing economic development for the town by the municipality.

WGH plugs itself into the local assemblage, one which was described above through having interesting history, culture, and traditions. They use the expressive and semiotic components of the local assemblage, and combine them with their vision of the town, and what it could be. For example, they offer engraving classes (a traditional practice from the area) where participants can engrave from photographs of the lagoon and the fisherman’s dwellings, or something else if they would like. They are very active on social media, always capturing a sunset or candid pictures of the lagoon. . The creative activities vary weekly and can include making paper, creating notebooks, painting, animation workshops, screen printing to name a few. The founder explained that when he was younger, creative activities were scarce, there was only football and he had no idea you could be an artist, a photographer, a graphic designer etc. Along with being creative and learning new skills, the workshops are aimed to foster collaboration. They try to pair older kids with younger, passing on the knowledge the older ones have learned in the classes. The hub also acts as a meeting point. While hosting different events and exhibitions, the different activities that take place more regularly act as an excuse for locals to meet and chat. It was very common for parents of kids to gather outside the hub on a Saturday with a coffee after they collected or dropped off their children. One day the founder described it as a cafe because of all the comings and goings. On some occasions people would stop by and discuss everyday matters, such as local politics, planning for the upcoming festival, or to give out about the available mezze options in the town. One of the CWS’ collaborator’s stressed the importance of the CWS as a meeting place for people to come together and collaborate to address the town’s problems -

*there’s no meaning to work separately. What we need is a centre, a centre to be altogether, to discuss and exchange ideas, because we have to think things, how all the guys here are working with minds, with hands, with everything, and it’s very important, and nobody by themselves can do it. We need to be altogether.*
                        (WG6, Interview)

The CWS provides this centre as a social infrastructure ( Klinenberg, 2018) for the town, along with providing creative activities that are also lacking. Through acting as a meeting point, it allows different people who would not normally cross paths to meet. Their activities address all ages, some engraving groups had participants ranging from 14 to 70. They have an oral history group that aims to retain oral traditions of the town, remembering specific people and events. They also collaborate with an ecologist and a local fisherman’s collective to provide an educational programme for children, working with several local schools.

These activities are provided to address the local needs of the community but are part of a bigger picture. They form part of a larger whole, an arrangement of desire, which, as shown before, is territorialised through the combination of different local components, such as local culture and natural resources. WGH has a vision of a future that it wants to produce - a flourishing town, where people take an active role in society, not *“waiting for the great mayor”* (WG1, interview) to come and fix things, but through small everyday actions they can construct a better future. Regarding their economic struggles, they see slow tourism as a solution. However, beyond the economic, they want a vibrant cultural life, people to care for and understand the environment, have local people engaged in activities outside their job and be involved in community activities. Through the existence of the CWS and its activities they offer a window into the future they desire to create – a community economy.

In CUA, we see a similar arrangement of desire. While they do not focus on local culture and traditions, they also make use of several material (e.g. technology) and expressive (e.g. open and collaborative ethos, ‘play’ mentality) to do so. While the local ‘economy’ is strong, they are lacking in some of the ‘social’ aspects of life. Despite local associations existing - music groups, a football club, photography, church groups etc, they were described by one CUA member as only having older people, closed and competitive. CUA is the opposite, promoting youth involvement, being open and collaborative.

Furthermore, the CWS has an alternative view on economy, and is more explicit regarding its political subjectivity, with the CUA co-op being a member-owned co-operative. Their impact report critiques the endless growth-and-innovation society we find ourselves in, and that CUA ‘ *challenges this through the creation of resonant connections between people, things and the world’* (CUA Impact report, 2020). The municipality is an essential component, without which the model of free open space would not function, as the municipality pays its bills and provides it with public infrastructure to use. This enabling context creates a free space for experimentation and ‘becoming otherwise’ ( Miller, 2019). As their co-founder stated:

“ *we have kind of research Lab. **..** a free space where it's not based on the normal ongoing system… to create ideas, maybe for the future. Maybe it is quite small, but maybe change something if we need.”*
                        (UA1, interview)

This subjectivity plays a key role in territorializing their assemblage, and broader societal change is much more present in their narratives rather than the focus on local development by WGH. However, they are not ideological, nor rigid politically and collaborate with actors they would otherwise criticize such as various municipal, state, and market actors (e.g. commercial banks). The social change they envision, is a collaborative over divisive, one where people are active in civil society, and not only focused on waged work, hence their suitability for transitions to post-capitalism ( Chatterton & Pusey, 2020). Their activities are open to anyone in the community to join, and they have a full schedule of activities running that can be accessed online – art classes, technology classes for the elderly, repair café, woodwork, climate action group. Most of the focus is ‘being-in-common’, without competition, the need to succeed or achieve anything, as one UA6 highlighted.

They have a long history collaboration with the school, something that was addressed as a problem for the area – both the type of school it is (a technical school) and that it is old. Through their connections with industry, CUA co-operative provided over twenty 3D printers to the school, which are now integrated into the curriculum. They also have co-operated with certain teachers, doing different projects related to technology and independent thinking, and run similar projects funded by the regional authority with younger kids in preschool. Like in WGH, the CWS’s activities serve as an entry point for individuals to get assembled which can give rise to further participation in the assemblage. For example, UA7 explained how he started by doing some workshops and then became a full member of the CUA co-op:

*we did some workshops with kids and videos… And it got aired on a local TV station for about two years, and I did some workshops with them. That was the first kind of thing we did. And then I just participated on some other workshops like it's nice or something and not blitzkneisser, but repair cafe. And then I started working at the CUA co-op.*
                        (UA7, Interview)

Moreover, both CWS do not just employ anyone but ensure they have the same mindset and perspective (the same coding) to ensure the territorialization of the CWS assemblage In WGH, before hiring the staff, they were asked to write a personal essay on the town and the hub, why they think it is important. One of the staff members mentioned how he felt the CWS’ goals are the same as his personal goals. In CUA, when looking to hire a new employee for the eGen, they put out an advertisement searching for someone “ *who is able to use a truck, able to work with wood material, who is open minded and able to have an open and mindful conversation with people.”* (UA5, Interview)

It appears that both CWS assemblages have a core of labour (waged, paid external collaborator, volunteer, reciprocal) composed of a number of ‘key individuals’ ( Neumeier, 2017; Richter & Christmann, 2023) that are at the centre of the CWS, or SI assemblage. These individuals take charge of certain groups and activities, for example the employed artists, the ecologist, art teacher, dance teacher, or member of the oral history group in Greece; or the art teacher, repair café member, member who gives art classes, or the member of the climate group, among others in Austria. Moreover, both CWS have two leading figures that act as gatekeepers of the assemblage. Both may be considered as social entrepreneurs, and have a considerable impact on the assemblage, providing it with many affects.

### 5.2. Changing social relations

In this section, we examine ‘changing social relations’, the second dimension of SI, considering how individual subjectivities are both re-configured and transformed into collective subjectivities through the CWS. Social relations and the subsequent increasing of affects and capacities towards community interests forms an essential part in the part of becoming post-capitalist subjects ( Gabriel & Sarmiento, 2020). Moreover, these actions form an affective relation, as they also provide the CWS’ with affects and help with its everyday reproduction.

***5.1.1. Desiring production and assisting with capacities.***

Both CWS’s act as nodes that give actors opportunities to affect and be affected, by providing them with resources, generally without charge, such as space, knowledge, materials, networks, equipment etc. that increases their capacities, and enables them to bring desires into reality, or in assemblage terms, desiring production. Part of their association comes from the ability of the CWS to provide meaning making, or axes of resonance ( Rosa, 2019) through non-alienated and significant relationships with the world and with others. Rosa (2019: 171) identifies resonance as a basic human capacity and need, and that all human desire, is a desire for resonance. It is this aspect that gives CUA’s motto – ‘CUA does not do anything, CUA makes possible’. This highlights how the CWS innovate not in an economic or technical sense but as *innovation in social relations*. They create and aid in the creation of new collaborations in places against the narrative that nothing is happening. We can regard this as SI practice, or outputs of SI processes ( Moulaert & Mehmood, 2020). It also highlights how the three dimensions of SI (addressing social needs, changing social relations and collective empowerment) are closely intertwined ( Moulaert & MacCallum, 2019). By addressing the needs of the local community (inertia in this case), they increase actors’ agency changing social relations and empower them with collective sense of identity.

One interesting example of this comes from an art teacher/artist, who first moved to Western Greece during COVID, as a substitute art teacher. He initially approached the CWS to meet local people, and once familiar with the CWS’ activities, collaborated with them for an engraving summer school, which evolved into a weekly workshop, and as an element in their environmental educational programme. The teacher noted the personal importance of these activities, mainly improving his skills and doing something meaningful. The CWS also gains new capacities through hosting new artistic activities, and from the atmosphere the teacher would bring, calling in frequently for small chats and gossip. The development of this affective relation also ran through the local community. From participating in the annual festival of the hub, it gave visibility to the art teacher, and he was approached by a local NGO that does artistic workshops with young kids and children with disabilities.

There were several other examples of the CWS collaborating with local people to help realize their desires, such as giving sound equipment to owners of a local tavern on their re-opening night, helping an elderly woman publish a children’s book and giving a young girl books, links and historical information about the town, in order for her to do a presentation to 150 people about a historical event, where the hub marketed the event, provided her with lights and sound equipment to make it happen. Regarding staff, it used to employ two artists; one of them left and the other could no longer be paid because of the end of a state funding programme. However, this affective relation was incredibly important for the CWS, for the town to retain two young artists, and for the artists themselves, who could have employment in their hometown, develop their careers as artists, and do meaningful work (e.g. with children, the environment). As WG11 stated, “ *[artist 1] and [artist 2] are kids that probably the town would have nothing to do if the hub did not exist”.* The CWS and the town gained two artists, who pass on their knowledge to young kids. The mutual benefit was highlighted when one artist independently took part in a prominent Greek animation festival, and the organizers came to the CWS do an animation workshop with young kids. Moreover, despite them leaving, the CWS is still interested in their professional and personal development, and they still collaborate on projects when they have the time and funding.

We see a similar picture in CUA, at the core of what they do is allowing people to follow their ideas and interests, helping people to find “ *what they really, really want”* to do. This happens through CUA’s model of free open space where people can encounter new activities and interesting people. Sometimes this happens to the extent that people can follow ideas into a funded project, generally from municipal, regional, or national funds. For example, the CWS has a climate action group that started meeting informally to create a discussion about renewable energy in the town, they have since carried out several municipal funded renewable energy projects in several public buildings. Another project between CUA, the school and the European Capital of Culture funds entailed schoolkids working with CUA in groups to realize their ideas (such as a film night with friends, hosted in CUA). On occasions an informal group forms into a more coherent body, for example a food co-operative was incubated there, also a company that makes 3D printers. On the individual level, CUA can unlock desires that otherwise would not have been possible living in a small town. One of their employees began his relationship with CUA as a teenager, taking part in a soldering workshop, He since has a degree in computer science and works on technology projects with the CUA co-operative. He credits CUA for him following this career path.

Here we can see the CWS’s acting as a relational tool, creating new relationships between locals. They act as a middle ground, assembling different actors, with material (e.g. equipment) and immaterial (e.g. funding, knowledge) components they previously would not have access to, that enables them to realize their own projects, achieving their own desires. And almost all these happen in the framework of diverse and community economies, with affective labour as a core codification and resource.

***5.1.2. From the individual to the affective community.***

SI theory tells us how SI processes can lead to collective empowerment by changing social relations from individual to community needs ( Gibson-Graham & Roelvink, 2009). From initially assembling individuals through their porous membrane, these individuals become subjects of the CWS assemblage, and enter new relations, forming part of the CWS’ affective community ( Zink, 2019). For example, WGH, as noted before, organizes an annual festival, with a focus on local culture, heritage, and traditions they create an affective arrangement that forms a collective identity. Given their lack of resources to employ the number of workers needed to plan and manage the festival, they make use of the affective labour of the local community to help them. This mainly consists of the core of labour that composes their everyday activities but also other (mainly younger) volunteers who return for the summer. Here we see the mixing of the various individuals and groups that help reproduce the CWS, whose roles can vary from simple administrative tasks to hosting workshops about local culture and traditions, or art exhibitions. The festival acts as a temporary arrangement of desire enacted, bringing together collective action and affective labour to create a window into what could be for the town, if everybody pooled together, highlighting what is possible in a diverse community economy.

In CUA, they also change social relations towards collective subjectivities through affective labour. The monthly repair café in CUA epitomizes this. The members of the group offer their labour, knowledge, and materials to the community for free, while they collaborate with other repair cafes nearby to share materials and ensure they don’t clash timetables. Their impact report states how these practices of repair *are intended to contribute to self-efficacy and self-empowerment, going beyond the relationship of consumption with objects*. Another interesting group was the ‘death café’ where people would meet up and talk about death, changing a typically individual and personal subject into a collective one –

*something I loved was the death café, where people talked about death for hours.. Just about the topic death. What do you think about death? What do you think about and how do you experience? What experience do you have with death and the setup was sitting around drinking coffee, eating cake and discussing death. I like the setup and the chance to talk about topics where you don't have the chance to talk about it at home with, the people you love.*
                        (UA7, Interview)

This collaborative ethos is also apparent with the many collaborators each CWS has. By being open, and not focused on profit, their ethos is reciprocated. As one CUA member stated, “ *if you're generous to people, normally people are generous the other way around”.* In CUA, they often receive donations of equipment that they can either use for parts for repair, or in the organization such as laptops, coffee machines, and kitchen utensils. These practices can manifest change at the individual level, forming new collective subjectivities, as people want to contribute to the community economy. UA6, formerly the chairwoman of the association, noted how being part of CUA affected her own ways of thinking, how old buildings can be used to benefit the community:

*And the thing is that the CUA philosophy changed my work within the community or is influencing how I see things in the community, like having a lost place or an unused house that we can do there something without money, and what to do in social life.*
                        (UA6, Interview)

Despite the organization of the various groups, there appears to be less mixing of the groups than as we saw in the WGH festival. However, individuals take part in multiple groups and roles in the association, for example the art workshop co-ordinator interviewed is also part of the ‘magic 5’ management team, the member of the climate group is also part of a free radio group in another CUA location, and the repair café member is also a paid employee of the eGen and takes part in another group with a technology focus.

More than just changing relations among their community, the CWS’s influence other larger assemblages with their open and collective way of doing things. We may understand how CWS’s affect other assemblages, through the concept of relations of exteriority, that each component part of the assemblage is autonomous, and can form part of other assemblages. Through collaboration with schools, and their existing relationships with the environmentalist and fishermen, they created a programme to address the lack of connection with locals and the natural environment, and the poor infrastructure of the public schools in Greece. Recognizing the exteriority of relations allows them to teach the schoolkids, inform them, and allow them to bring their knowledge into other assemblages, such as their families, and other groups they are part of, something Avdikos & Pettas (2021) refer to as diffusion of the CWS collaborative culture to broader circuits.

Working with schools, the fishermen co-op and the ecologist, educate the public about the local environment and its importance for the town, that it’s not a distant far away thing or merely a resource to be exploited. They work with the fishing co-operative, who fish seasonally, but the rest of the year still must operate the ‘Divari’ (traditional fishing structure) to maintain the lagoon, keeping it clean and refreshing it with enough clean water. The ecologist explained how only the co-operative does this maintenance work, and there is an issue with private fishermen trying to win fishing rights, who only fish for profit rather than working with the lagoon and the environment. Through this education, WGH reframes the relationship between the ‘hegemonic trio’ of economy-society-environment ( Miller, 2019), not as separate spheres of life, but as interconnected and contingent on one another with a collection of human and non-human actants that co-exist and co-depend in the same geographical space.

As already mentioned, CUA has a long-standing relationship with both the municipality and the local school, spanning over 10 years. Their relationship with the municipality has the agreement that the municipality will pay for the space and its maintenance, but will not interfere with it in any way, that it is free of party politics, and they should not expect any kind of output or results. The CWS focus is on the local community, and therefore, like WGH, the value it creates is not for the CWS, but for the town. In WGH, while initially having an antagonistic relationship with the municipality, it appears to be changing. With a new mayor last year, he approached the CWS to organize an open public consultation regarding the town’s development, for all citizens to attend. Adhering to the exteriority of relations allows the CWS’ to penetrate other assemblages, and to ‘infiltrate’ other more strictly coded and highly territorialized assemblages, such as schools and municipalities, while maintaining their own autonomy and not adhering to their codes. In Austria, the school also benefits from CUA’s existence, through shared projects, a summer school and the donation of 3D printers. The local community also benefits from the re-coding of property to the community through the opening of public property outside of school hours. For example, a wood turning group uses the woodwork room. While initially difficult to understand, one teacher explained how it changed their own perceptions about the school infrastructure:

*you know it's only for the short period when it's being used, then actually for the majority of the day it’s completely empty…. this was this was new for us, but some ones come in my arts room. It's not my arts room, but I thought about it. Why do I react in this way?*

Therefore, in both CUA and WGH, along with connecting with local people and organizations they also assist institutional assemblages such as schools and the municipalities, to varying degrees. To summarize the findings, the CWS assemblages assemble material, immaterial and semiotic components and address needs of individuals by providing them with meaningful activities and offering resources that allows them to fulfil their desires and increase their capacities. Consequently, individuals are assembled as part of the affective community of the CWS assemblage that helps with its reproduction, through specific temporal enactments such as a festival, repair café, workshops and educational programmes. These temporal assemblages create socio-material networks composed of ethical relations among actors based on collaboration, reciprocity, care, and ecology through a variety of transactions (market, gifting, reciprocal exchange, donations), property (private, publicly owned) and labour (waged, self-employed, volunteer). In both cases, there exists a few key individuals who act as leaders within both assemblages and whose affects have greater impact. These affects and the value generated by the CWS assemblages accrue to the local area as well as the CWS.

## 6. Discussion: the transformative potential of Collaborative Workspaces

So far, the paper has discussed the role of CWS as initiators and facilitators of SI processes in two community led CWS in two rural areas in Austria and Greece. The findings reveal that CWS play a significant role in addressing social needs and facilitating grassroots SI practices and processes, creating affective relations that contribute to the transformation of individual to collective subjectivities, thus answering the first research question “ *How do rural collaborative workspaces engage in social innovation processes?”*. The second research question *“To what extent can these SI processes be considered to have transformative potential for society?”* and the third more theoretical research question “ *How can diverse and community economies and assemblage thinking inform transformative social innovation theory?”* will be answered in this section. As elaborated upon in the literature review, both DCE and AT offer tools for thinking about transformations towards post-capitalism and post-growth realities, through their anti-essentialist, post-structuralist ontology. DCE offers a discursive shift of our understanding of the economy to more-than-capitalist and non-capitalist enterprise, labour, transactions, property and finance relations, while AT offers an analytical framework coupled as an ontological and methodological tool for rethinking spatial forms.

While very different socio-economic contexts, the processes appear quite similar. In both CWS assemblages the multiplicity of relations is clear from the municipally funded space of CUA, volunteer/affective labour apparent throughout both CWS assemblage’s activities, the self-employed labour through the external collaborators of WGH, and their financing from EU and state projects, along with financial and material donations. The main contrast is the ease of CUA in accessing resources from the municipality or local government compared with WGH having to rely a lot more on their own actions to raise money through market actions, funding programs or community donations, which adds an element of precarity to their assemblage and the risk of mission drift. This highlights that within a DCE framework, there still exist elements of capitalist logic and innovation. Part of WGH’s vision is to create economic growth and employment, to stimulate both supply and demand and attract wealthy tourists to spend money in their town. They have waged employees, and engage in competitive funding tendering, market exchange (tourism, sale of products). These elements appear more apparent in WGH than in CUA, because of the dire economic circumstances of their town, the need for them to pay for their property and earn a living through the CWS, whereas CUA has a supportive context through the ease of access to funding through both local and regional government. The lack of a supportive environment means the CWS must trade on the market and spend time applying for funding to earn some income to keep it afloat economically despite its successful model of solidarity donations from locals. This constant chasing of economic income can leave them with less time for community activities, struggle to permanently employ staff and can lead to internal tensions, for example one of the artists left because of the pressure he felt from the founder. Moreover, although both CWS offer the opportunity for desiring production, it is not entirely free, and appears somewhat overcoded by both founders, albeit to less of a degree in Austria. In WGH, almost everything refers back to the local town and culture, while in CUA there is a specific technology focus, which is the main interest of the founder. While both founders have a significant influence on the assemblage, CUA has much more rhizomatic character, as a co-operative but also in the association. Their network of associations spread across several countries share this character, autonomously governed and sharing the same principles. WGH, while porous with the community, the organization has a very hierarchical character, which most likely occurs because of the difficult socio-economic context and the reliance on the founder to drive the assemblage forward constantly. However, it does have a rhizome-like interaction with its external collaborators, which could allow for a similar spread of SI in the region, as seen in CUA.

The fact that WGH engages more with capitalism does not rid it of transformative potential. Firstly, we need to acknowledge the very different socio-economic realities that exist in Northern and Southern Europe, and what this means when thinking about societal transformations. Post-growth thinking acknowledges these inequalities, that sometimes an increase in material wellbeing and prosperity is required in the Global South and disadvantaged populations in the Global North ( Schmid, 2022). Moreover, by drawing from assemblage and diverse and community economies thinking, it allows us to move beyond essentialized forms, whereby “ *capitalism becomes just one particular set of economic relations situated in a vast sea of economic activity”* ( Gibson-Graham, 2008: 68). This is clear in both cases, from the vast array of labour, transactions, property and finance apparent in both CWS assemblages. Moreover, assemblage thinking does not have us to question whether these assemblages are transformative or not, but what is their potential for social transformation. When thinking with assemblages, we do not ask what a body is, but what can a body do? ( Buchanan, 1997). Therefore, going beyond the material conditions of both cases, it is clear that both CWS assemblages address social needs and engage in the two levels of changing social relations: desiring production (praxis) and changing individual to collective subjectivities, these processes have emancipatory potential. Through offering resonant experiences and meaningful connection and allowing people to engage in free-flowing desire production, it destabilizes what Deleuze and Guattari describe as the machinic unconscious, unlocking a revolution of micropolitical becoming in those that encounter the CWS’s. Rather than being subjected to machinic enslavement, inertia and alienation, the CWS’s offer liberation from the circuits of the machines at play in both localities, be it through the ‘left-behindness’ in Greece or lack of purpose in Austria ( Harvey, 2020; Rosa, 2019). Moreover, through creating affective communities, this affective labour has transformative potential, as it creates collective subjectivities, social networks, community, and biopower from below ( Hardt, 1999). These reconfigurations enable the creation of what Gibson-Graham (2006) call ‘intentional economies’ or ‘real utopias’ as Wright emphasised ( 2010). Both represent open ended post-capitalist realities, by facilitating pluriversality ( Escobar, 2021) or economic diversity ( Gibson-Graham, 2006), working towards creating many different worlds, rather than the perceived dominant capitalist world, destabilizing its perceived hegemony.

Rather than providing an exact post-capitalist strategy, the CWS’ provide a “ *multitude of possibilities of what could come after, as well as building daily competences to leverage social change.”* ( Chatterton, 2016: 405). However, thinking about post-capitalism through DCE, ‘ after’ does not imply a clean break from the ‘capitalist system’ and instilling a better system, but by recognizing the capitalist alternatives in the here and now ( Miller, 2019; Schmid, 2022). To help conceptualize how this occurs, we return to Wright’s (2010) three pathways towards social transformation – *ruptural, interstitial and symbiotic.* Wright gives examples of interstitial transformations such as various forms of co-ops, workers councils, community based social economy organizations etc., which are also common examples of SI. Such initiatives offer viable alternatives to state and capitalist ways of organizing, however, are often critiqued as operating in isolation, whereby they do not offer a real viable alternative, but merely as heterotopias that operate within ‘niche’ or ‘protective spaces’ ( Smith & Raven, 2012). This is what we conceptualize as our first level of transformation, which we can clearly see as the two CWS’s work towards satisfying community needs and building community economies, through desiring production and building affective relations.

Wright differentiates from interstitial to symbiotic transformations from their orientation towards the state, which they use as a mediating body to reduce capitalist power, with the main example given as class compromise, where workers and capitalist firms negotiate through the state over workers’ rights, pay etc. We would like to offer a different conceptualization of symbiotic transformation, as a second stage of interstitial transformation diffusion, as they influence other spatiotemporal arrangements. This may initially be in antagonism, and lead to their practices being copied/replicated by state or municipal organizations, or it may mean these institutions recognizing and supporting interstitial transformations as important social economy actors. In both cases we also observe this level of transformation, with long term institutional collaboration of CUA and the municipality and more recently in WGH. Moreover, both CWS also offer their resources and capacities to local actors such as schools, local businesses and other market entities, NGOs, and other local associations, blending DCE economies with the market economy. While interstitial transformation appears to be performed inside the CWS, symbiotic transformation happens outside the CWS, influencing other entities and the state to experience the alternative socio-economic arrangements, through the DCE framework that CWS’s assemblages perform. Within SI literature, symbiotic transformation is often used to describe the transformative aspect of SI as bottom-linked SI ( Haxeltine *et al.*, 2016; Moulaert & MacCallum, 2019) that *“develops when citizens' collective initiatives result in agreements with local institutions that enable and sustain such initiatives through sound, regulated and lasting practices”* ( Garcia *et al.*, 2015: 93). This symbiotic approach is key for SI’s transformative potential to move beyond their space in the margins. However, SI being recognized by institutions does not equate to the neoliberal rhetoric by governments over the last twenty years (for example see Fougère *et al.*, 2017 for a full discussion), something that *“limits social innovation to marginal change”* ( Bartels, 2017: 3802). Initiatives need to be given resources and power rather than just responsibility ( Bragaglia, 2021).

**Figure 1.**
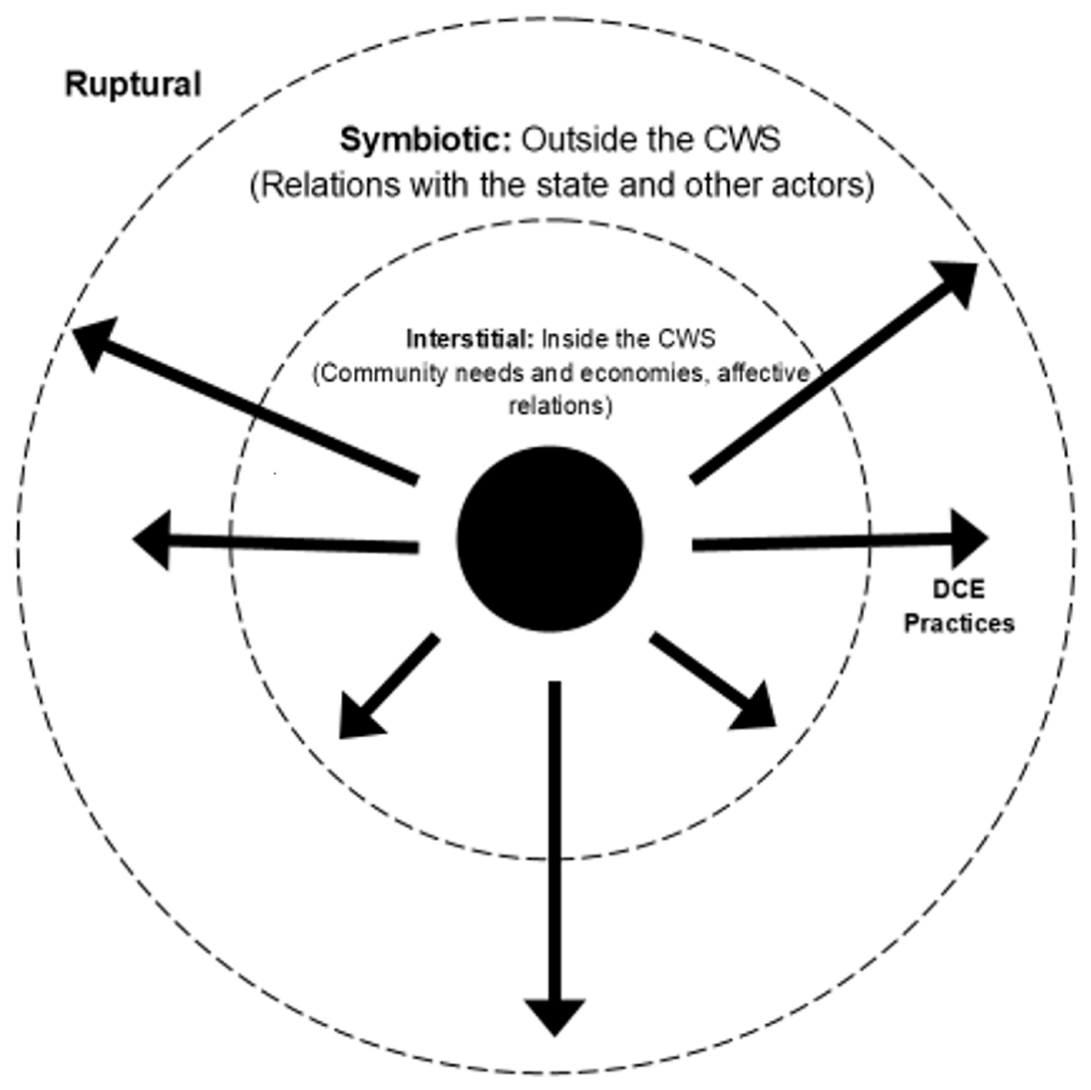
How Diverse and Community Economay practices diffuse throughout the three levels of transformation *to create a transformative change, to(wards) post-capitalism.*

As shown in Figure 1, it is important to not think of the stages as linear steps and stages, and to note there is a degree of non-linearity as DCE practices are performed and practiced, moving from the edifices and margins as interstitial transformations, to piercing the institutional dimension, back to interstitial and moving beyond the symbiotic ( Chatterton, 2016). Regarding the ruptural stage, we envisage DCE practices spreading in a form of contagion until they reach a saturation point of post-capitalist becoming, signifying not necessarily a straight clean break from capitalism to post-capitalism, but a revolution of becoming, as different aspects of life become recoded and DCE are heavily territorialized and enacted in society. Both cases do not reflect this level of transformation (yet, at least), but an example of such a ‘real utopia’ would be the widely documented case of Mondragon in the Basque country, with the co-operative model completely institutionalized in the region. While as an example it is highly contextual and non-replicable, it gives an example of possibility ( Gibson-Graham, 2008). We cannot state this is the case for either case study, both remain still in their symbiotic stage. The passage to a ruptural transformation requires CWS to be supported by various local and extra-local actors to be acknowledged as the only alternative and post-capitalist solution, where DCE can become the dominant mode of production and gain a hegemonic place in society.

## 7. Conclusion

The paper discussed the role of CWS as initiators and facilitators of SI processes in two community led CWS in two rural areas in Austria and Greece. The research methodology employed a qualitative case study approach, utilizing participant observation and semi-structured interviews to explore the multiple sets of relations found in these rural CWS, aiming to understand how these arrangements cover local needs and foster new social relations. The findings reveal that CWS play a significant role in addressing social needs and facilitating grassroots SI practices. CWS seem to provide a site where the unmet needs of the local people are negotiated, redefined and are covered in a co-creative and collective manner. This is happening in a DCE framework, where affective labour mostly performs. They provide opportunities for actors to affect and be affected, increasing their agency and assisting them with their own desires by providing resources such as space, knowledge, materials, networks, and equipment.

On the individual level, these actions have emancipatory potential through freeing desiring production and creating non-alienated and significant relationships with the world and with others. These affective relations also extend to the larger community in a symbiotic manner, creating positive affects for the town and fostering collective action and affective labor to create a window into what could be for the community and help to develop collective subjectivities. From this interstitial level, these processes also enable symbiotic transformations as they 'infiltrate' other more strictly coded and highly territorialized assemblages, such as schools and municipalities, while maintaining their autonomy. These processes give potential for a multiplicity of alternative development pathways, from interstitial, symbiotic and potentially ruptural transformative potential.

The main aim of this paper was to provide a theoretical framework of assemblage thinking and diverse and community economies to firstly understand how CWS engage in SI processes, and secondly to unpack how these processes may have potential for societal transformation. These concepts, through their anti-essentialist approach allowed us to move beyond the inherit binaries existing in both CWS and SI literature. Firstly, they allowed us to see beyond the individual entrepreneur at the heart of the assemblage and understand the processes of individual and collective empowerment that occur. This is important for both SI and CWS theory, especially as much of the literature focuses upon binary discourses of either of neoliberal individual ‘changemaker’ entrepreneurs facilitating and accelerating social change, or community-based collective endeavors ( Bandinelli & Arvidsson, 2013; Gandini & Cossu, 2021; Montgomery, 2016). An anti-essentialist approach allows us to see beyond these divides and understand CWS as syntopias rather than absolute heterotopias, whereby both capitalist and non-capitalist subjectivities can co-exist while not opposing the other, and potential for transformation may already be latent within ( Vidaillet & Bousalham, 2020). Future research could further explore these tensions and contradictions inherit to SI, but also the wide range of SI’s that exist, from the conformist and reformist capitalist ones ( Liodaki, 2024) to radical anti-capitalist SI’s ( Apostolopoulou *et al.*, 2022) and how they may interact with Wright’s three stages of transformation. Furthermore, the theoretical framework questions the assumption that SI emerges from a lack of services and unmet social needs ( Moulaert & MacCallum, 2019). When viewed through AT, it appears that SI emerges from a desire to change social relations, often towards more ethical and ecologically sustainable arrangements, and the CWS managed to tap into that desire, and addressing social needs in the process.

Finally, there is an urban bias within SI research, despite the growing body of rural SI literature. Some of this work recognizes the plurality of economic relations, such as Olmedo & O'Shaughnessy (2023) through a Polyanian framework. Consequently, there is a need for comparison between rural and urban areas and determine if there is indeed a difference in the character of SI we see, and if so, how can the understandings of one benefit the other. Within the context of social-ecological crises, it alters the position of urban agglomerations vis-à-vis the rural. Notions of progress and development have been linked with urban agglomerations, seen as drivers of national economies through innovation, with the rural seen as traditional, backward, lacking – thus implying a need to transformation towards being more urban ( Shucksmith *et al.*, 2009). However, in light of the polycrisis, we cannot solve modern problems, with the very categories that created them – growth, progress, competition, urban economic dynamism ( Escobar, 2021). Instead, we must focus on those livelihoods that do not fully assimilate to the supposedly dominant capitalist economy, which is often clearer in rural areas where social relations may be more embedded with nature and communal life, if not part of the same habitat ( Miller, 2019). Rural-urban comparisons can open new questions thinking about SI, such as what territorializes the assemblages of urban SI? Does more intense financialization of space, as observed in urban centres, make urban SIs lean more towards the market? Or what are their practices of subverting this? Moreover, what could rural SI learn from urban examples, for example the new municipalist experiments in cities such as Barcelona and Madrid ( Thompson, 2021). Such questions emerge when comparing similarities while being aware of differences, as was the case with the comparison with Greece and Austria. Moreover, assemblage thinking can help with this, as it did with this paper, with its purpose not to develop a homogenous field manual for post-capitalist transformation, but to deconstruct the socially innovative assemblages and their processes of (de/re) composition, and understand how these processes and their outcomes in two very different contexts share some similarities, why that is the case, and what conclusions can we draw regarding their transformative potential.

## Ethics and consent

### Consent

Interview Participants were given an information sheet about the study and were required to sign a consent form prior to being interviewed. An ethical approval committee at Panteion University approved the study (Protocol Number: 44/ 30-9-2022). An example of the information sheet and consent form can be found above in the ‘extended data’ section.

Regarding participatory observation, the researcher made his identity as a researcher known to the users of the CWS by contacting the CWS in advance via email, requesting that they inform relevant stakeholders that there will be a researcher present for the given time period, and to notify them should they not consent to participate in the study to contact the researcher through contact details provided. When present on site, the researcher made his identity as a researcher known to the those participating in the CWS’ activities, and verbal consent was required because of the short time duration of their visits. Moreover, although minors took part in some workshops (as stated in Section 5.1.), the participants in the workshops were not interviewed or subjects of participant observation, and therefore it did not require consent.

## Data Availability

Due to ethical restrictions on data sharing should any reader or reviewer request data to be made available, small sections of de-identified quotes may be available on request by emailing the corresponding author. ( colmstockdale@panteion.gr) Figshare: Colm_Stockdale_Information_Consent_Form. Stockdale (2024a). Colm_Stockdale_Information_Consent_Form.docx. figshare. Online resource. https://doi.org/10.6084/m9.figshare.26356546.v1 *This project contains the following underlying data:* The information sheet and consent form I used for my study to inform participants about the study and obtain their consent for participation. Data is available under the terms of the Creative Commons Attribution 4.0 International license (CC-BY 4.0) ( https://creativecommons.org/licenses/by/4.0/). Figshare: Table 2 Regional Demographics, Stockdale (2024b). Table 2 Regional Demographics. figshare. Dataset. https://doi.org/10.6084/m9.figshare.26356738.v1 *This project contains the following underlying data:* A table comparing regional demographics between the region of Western Greece and Upper Austria under indicators such as Population, GDP per Capita at current prices, Employment rate %, and % of persons at risk of poverty or social exclusion. Data is available under the terms of the Creative Commons Attribution 4.0 International license (CC-BY 4.0) ( https://creativecommons.org/licenses/by/4.0/). Figshare: Interview Protocol. Stockdale (2024c). Interview Protocol. figshare. Online resource. https://doi.org/10.6084/m9.figshare.26370316.v1 *This project contains the following underlying data:* The document contains the interview protocol for the study. This includes the research question and sample interview questions asked to interview participants. Data is available under the terms of the Creative Commons Attribution 4.0 International license (CC-BY 4.0) ( https://creativecommons.org/licenses/by/4.0/). Figshare: Research Protocol. Stockdale (2024d). Research Protocol. figshare. Online resource. https://doi.org/10.6084/m9.figshare.26370331.v1 *This project contains the following underlying data:* This document contains the research protocol used in the study. It contains the timeline for fieldwork and the Data collection procedures (data protection, identification of data sources). Data is available under the terms of the Creative Commons Attribution 4.0 International license (CC-BY 4.0) ( https://creativecommons.org/licenses/by/4.0/).
