# Supplementary material for: Transformative social innovation and rural collaborative workspaces: assembling community economies in Austria and Greece
Source: Open Res Eur. 2025 Jan 20;4:205. Originally published 2024 Sep 19. [Version 2] doi: 10.12688/openreseurope.18007.2 (PMC11757924; doi:10.12688/openreseurope.18007.2)
Supplement: Peer review history

# Reviewer report: Reviewer response for version 2

**DOI:** 10.21956/openreseurope.20821.r49950

**Published:** 2025-05-25

**Authors:** Gary Bosworth

**Affiliations:** 1 Northumbria University, Newcastle upon Tyne, England, UK

Thank you for your detailed comments and clarifications in response to my first review. And sincere apologies for the very long delay in completing this second round review. 

 The purpose and focus are now very clear and the literature review section on Social Innovation captures an impressive amount of information in a relatively succinct section. The variety of new collaborative workspaces comes through clearly and it is logical to assume that the nature of social innovation in each case cold be quite different – both in terms of social organisation and in terms of social forms of participation.

 I am not a big fan of assemblage theory, and personally I might prefer to look at functionality and networks as two separate aspects of the CWS.  However, the way that you are able to use this as a strong thread throughout the analysis of the findings suggests that you have hit upon an approach that does add to our understanding of CWS, their different features and their different and overlaying networks of connections. I particularly like the way that you bring the discussion back to social innovation and use the assemblages approach to help explain how social innovation can (and in these types of situations perhaps should?) be a collective activity.

 Having previously commented that the contribution of the paper was a little “underwhelming”, I find the deeper theorization in the paper demonstrates how the research is contributing to larger debates in rural development, collaborative workspaces and social innovation literature. The data remains a strength and the use of quotations an explanation of the cases are strong features of the paper that reassure me of the robustness of the claims made. It will be interesting to see how the CWS landscape, particularly, in rural areas, continues to evolve but this paper certainly provides a solid basis for future researchers to refer to when analysing the next phase of evolution in collaborative working.

If applicable, is the statistical analysis and its interpretation appropriate?

Not applicable

Is the study design appropriate and is the work technically sound?

Yes

Is the work clearly and accurately presented and does it engage with the current literature?

Yes

Are the conclusions drawn adequately supported by the results?

Yes

Are sufficient details of methods and analysis provided to allow replication by others?

Partly

Are all the source data and materials underlying the results available?

Partly

Reviewer Expertise:

NA

I confirm that I have read this submission and believe that I have an appropriate level of expertise to confirm that it is of an acceptable scientific standard.

# Reviewer report: Reviewer response for version 2

**DOI:** 10.21956/openreseurope.20821.r49952

**Published:** 2025-02-13

**Authors:** Athina Arampatzi

**Affiliations:** 1 Regional Development Institute, Panteion University of Social and Political Sciences (Ringgold ID: 69001), Athens, Attica, Greece

Thank you very much for addressing my suggestions in such a thorough manner. I believe that the article has improved a lot and opens up several ways for further enquiry.

 Kind regards,

 Athina

If applicable, is the statistical analysis and its interpretation appropriate?

Not applicable

Is the study design appropriate and is the work technically sound?

Yes

Is the work clearly and accurately presented and does it engage with the current literature?

Partly

Are the conclusions drawn adequately supported by the results?

Partly

Are sufficient details of methods and analysis provided to allow replication by others?

Yes

Are all the source data and materials underlying the results available?

Yes

Reviewer Expertise:

urban studies, urban geography, social innovation, urban governance, social solidarity economy, urban commons, social movements

I confirm that I have read this submission and believe that I have an appropriate level of expertise to confirm that it is of an acceptable scientific standard.

# Reviewer report: Reviewer response for version 2

**DOI:** 10.21956/openreseurope.20821.r49951

**Published:** 2025-01-29

**Authors:** Ignasi Capdevila

**Affiliations:** 1 Paris School of Business, Paris, France

The authors have satisfactorily addressed my comments on the earlier version. I accept the changes and believe the manuscript is ready for publication.

If applicable, is the statistical analysis and its interpretation appropriate?

Not applicable

Is the study design appropriate and is the work technically sound?

Yes

Is the work clearly and accurately presented and does it engage with the current literature?

Partly

Are the conclusions drawn adequately supported by the results?

Partly

Are sufficient details of methods and analysis provided to allow replication by others?

Partly

Are all the source data and materials underlying the results available?

Yes

Reviewer Expertise:

Qualitative methods

I confirm that I have read this submission and believe that I have an appropriate level of expertise to confirm that it is of an acceptable scientific standard.

# Reviewer report: Reviewer response for version 2

**DOI:** 10.21956/openreseurope.20821.r49949

**Published:** 2025-01-23

**Authors:** Bettina Bock

**Affiliations:** 1 University of Groningen, Groningen, The Netherlands; 2 Social Sciences, Wageningen University & Research Rural Sociology Group, Wageningen, Gelderland, Netherlands Antilles

I am happy with the changes made in response to my comments. in my view the paper is now ready to be accepted.

If applicable, is the statistical analysis and its interpretation appropriate?

Not applicable

Is the study design appropriate and is the work technically sound?

Yes

Is the work clearly and accurately presented and does it engage with the current literature?

Yes

Are the conclusions drawn adequately supported by the results?

Yes

Are sufficient details of methods and analysis provided to allow replication by others?

Yes

Are all the source data and materials underlying the results available?

Yes

Reviewer Expertise:

rural development, social innovation, peripheralisation, community initiatives

I confirm that I have read this submission and believe that I have an appropriate level of expertise to confirm that it is of an acceptable scientific standard.

# Reviewer report: Reviewer response for version 1

**DOI:** 10.21956/openreseurope.19462.r44830

**Published:** 2024-10-25

**Authors:** Ignasi Capdevila

**Affiliations:** 1 Paris School of Business, Paris, France

Introduction and Framing of the Research Question

 The article would benefit from a stronger framing of its research purpose and contributions within the introduction. Specifically, the authors should clearly articulate the research question early on to orient the reader toward the study’s specific aims and relevance. A more explicit statement of the research gap would enhance understanding of why this study’s focus on rural collaborative workspaces (CWS) and social innovation (SI) is both timely and necessary. To further contextualize this, the introduction could summarize existing assumptions within SI and CWS research, setting the stage for why these topics merit investigation together and framing CWS as potential vehicles for community empowerment in rural economies. A sharper outline of these elements will help readers recognize the paper’s unique position within the literature and its potential contributions to SI and rural economy discourse.

 Literature Review 

 The literature review would benefit from a more cohesive structure that clarifies the logical progression between sections. As it stands, there are limited transitions, which can make it difficult for readers to follow the development of the argument. To enhance readability, the review could begin by briefly covering the foundational literature on SI, followed by an exploration of CWS-specific studies, before focusing on the intersection of SI and CWS as a distinct research area. This organization would help clarify the gap the authors are addressing and lead naturally into the statement of the research question. Additionally, the analytical framework of assemblage theory could then be introduced as a means to address this question, explaining how the chosen theoretical perspective provides a lens through which to interpret the transformative potential of rural CWS. This restructuring would improve the paper’s clarity and better situate the study within existing scholarship.

 Methodology

 While the authors employ a qualitative comparative case study design, further detail regarding the data collection and analysis would enhance the methodology section. For example, more in-depth descriptions of data coding, the interview protocol, and any analytical software or techniques used would strengthen the study’s rigor and replicability. Moreover, the section discussing the localities is somewhat general and does not provide enough specific context to deepen understanding of how these distinct settings might shape or limit the CWS' potential impact. Since the paper aims to offer a comparative analysis, a justification for selecting Greece and Austria as cases is crucial. Expanding on why these two cases were chosen and how their specific socio-economic conditions relate to the research question would provide readers with a stronger foundation for interpreting the findings.

 Results and Discussion

 To improve clarity, the authors should more explicitly delineate the results and discussion sections. In its current form, there is some blending between the two, with references to the literature appearing in the results section and data quotes embedded within the discussion. This crossover risks confusing readers about where empirical observations end and theoretical interpretation begins. To address this, the authors could clarify the data structure in the results, using clear subheadings and more direct data quotes to support their findings. For example, using a "show, don’t tell" approach would enhance transparency, allowing readers to directly see the data backing each claim and strengthening the study’s credibility. In the discussion, removing direct data quotes and instead focusing on theoretical analysis and implications would reinforce the interpretive flow, making it easier for readers to understand the study’s contribution to broader SI and community economy literature.

 The discussion section could be strengthened by reinforcing key concepts introduced in the literature review. Some theoretical terms, such as Diverse and Community Economies (DCE), are used in the discussion without being sufficiently elaborated upon in the earlier review, creating a disconnect between sections. A brief but clear presentation of these concepts in the literature review would provide readers with a stronger grounding and allow the discussion to delve into these frameworks more seamlessly. Additionally, referencing back to the research question and explicitly connecting findings to the theoretical concepts of assemblage and community economies would improve coherence and underscore the study’s contributions to these fields.

 The methodology presents the two case studies as contrasting examples; however, the results section does not fully explore how these cases respond differently (or similarly) to the research question. A more focused comparative analysis would clarify whether the observed differences in CWS functionality or outcomes are due to local cultural and economic factors, or if they share common transformative elements despite contextual variations. Bringing these contrasts to the forefront would enrich the study’s insights into how CWS may function under varying conditions and provide a more comprehensive understanding of their potential across diverse rural settings.

 In sum, the paper offers valuable insights into the transformative potential of rural CWS through a theoretically informed case study approach. However, the paper would benefit from improvements in structuring the literature review, clarifying the methodology, separating results from discussion, reinforcing theoretical concepts in line with the discussion, and enhancing readability. These refinements would strengthen the paper’s academic rigor and readability, reinforcing its contribution to the discourse on SI, CWS, and post-capitalist community economies.

 Minor comments: 

- Correct typographical errors, e.g., “Western greece” should be “Western Greece” (p.6).
- Avoid acronyms in titles (e.g., replace "CWS" with the full term "Collaborative Workspaces").
- Define any acronyms when first introduced, and avoid overuse to enhance readability. Specifically: Clarify "MBL" on p.8, "DCE" on p.12, and "CE" on p.13.
- Eliminate redundant phrasing:  "For example, For example" (p.10)

If applicable, is the statistical analysis and its interpretation appropriate?

Not applicable

Is the study design appropriate and is the work technically sound?

Yes

Is the work clearly and accurately presented and does it engage with the current literature?

Partly

Are the conclusions drawn adequately supported by the results?

Partly

Are sufficient details of methods and analysis provided to allow replication by others?

Partly

Are all the source data and materials underlying the results available?

Yes

Reviewer Expertise:

Qualitative methods

I confirm that I have read this submission and believe that I have an appropriate level of expertise to confirm that it is of an acceptable scientific standard, however I have significant reservations, as outlined above.

# Response

**Published:** 2024-12-19

**Authors:** Colm Stockdale

**Affiliations:** Department of Economic and Regional Development, Panteion University of Social and Political Sciences, Athens, Attica, Greece

Thank you for your insightful comments. The introduction has been reworked accordingly to articulate the research purpose.  The organization of the text has been re-worked to accommodate this. With a separate literature review (Section 2) and conceptual framework (Section 3).  The methodology section has been updated to reflect this comment, regarding data coding, the interview protocol, and the analytical software used. Moreover, a section has been added that has more in-depth information regarding the contexts and how they relate to the research question.  The results section has been amended accordingly. There are several new quotes in the results section, and subheadings have been fixed. Quotes have been removed from the discussion section, which has also been amended to allow for more reflection on the research questions and the contribution of the study. Moreover, the concepts are presented more clearly in the literature review and conceptual framework sections. The discussion section also now reflects the comments regarding the different contexts. The minor comments have also been addressed.

# Reviewer report: Reviewer response for version 1

**DOI:** 10.21956/openreseurope.19462.r44823

**Published:** 2024-10-21

**Authors:** Athina Arampatzi

**Affiliations:** 1 Regional Development Institute, Panteion University of Social and Political Sciences (Ringgold ID: 69001), Athens, Attica, Greece

Dear authors,

 The manuscript deals in a very engaged way with the timely topic of the ongoing and fast development of collaborative work spaces (CWS), by exploring two cases of rural CWS in Greece and Austria. Through an engagement with diverse concepts and literature, it seeks to elicit the transformative potential of CWS for local communities and, by extension, their impact on social change more broadly.

 The manuscript is clearly presented in terms of its goals, aimed contribution to the literature and the analysis of its empirical material. The research design described is appropriate and the work carried out is sound, in terms of both fieldwork and analysis of research data. The methodological approach is also sound, while the necessary information on source data and results are available to the readers. Finally, the conclusions make an interesting attempt to bring forward the key arguments raised and outline relevant limitations and future research pathways.

 Having said these, the manuscript, while overall comprehensive, could benefit from a clearer and more precise delineation of its conceptual framework, to facilitate even further the development of the analysis and the conclusions sections, and stress its original contribution to the field of study.

 Here are some suggestions that could work towards these directions:

  

1. The work carried out holds, I believe, strong originality in terms of discussing CWS and SI in rural contexts, as opposed to most literature focusing on urban ones. This needs to be stressed throughout and comparative lines need be drawn and fleshed out in the discussion and conclusions sections (as well discussed in the comparative lens used between two different contexts of southern and northern Europe). For example, what differences there exist in terms of transformative potential of CWS in rural vs urban contexts based on the research? Are there specific lessons to be learned from this comparison?
2. There is a plethora of concepts employed, which, eventually, in my opinion, blur the core arguments to be delivered, and this is also reflected on the manuscript’s title. While each one has its own merit, taken all together might not work in favor of the manuscript’s goals. If, for instance, the core question posed is “how do local assemblages of CWS contribute alternative forms of community economies and/or transformative forms of social innovation beyond mainstream local development” the conceptual framework to be chosen towards this end might need to be more focused and specifically deal with this (or others) question as a guiding direction of the manuscript. Here another issue pops up: could assemblage thinking work better on a more methodological level to trace all the qualities, capacities, actors, networks, their relations, desires, affects, cultural trajectories etc. of such local community formations?
3. Discussion: I am a bit unsure whether Wright’s approach works here to unpack, compare and expand on the manuscript’s main contribution, which, again, I believe lies on the rural vs urban comparison.
4. Conclusions: the authors might want to highlight and expand on the main contribution of the manuscript, e.g. what does the shift from individual to collective empowerment observed in rural areas contribute to mainstream literature on urban contexts? Can we rethink the idea of transformative SI in this regard? If an institutionalization of informal activity is deemed necessary for CWS to develop in rural areas, what would this mean in relation to cities? And also, maybe expand on the limitations of gatekeepers/hierarchical positions within local assemblages, and their horizontal rhizomatic character.

Hopefully the above suggestions provide some useful directions to ameliorate the manuscript and strengthen its core arguments.

 Thank you for sharing your very interesting work

 Kind regards,

 Athina Arampatzi

If applicable, is the statistical analysis and its interpretation appropriate?

Not applicable

Is the study design appropriate and is the work technically sound?

Yes

Is the work clearly and accurately presented and does it engage with the current literature?

Partly

Are the conclusions drawn adequately supported by the results?

Partly

Are sufficient details of methods and analysis provided to allow replication by others?

Yes

Are all the source data and materials underlying the results available?

Yes

Reviewer Expertise:

urban studies, urban geography, social innovation, urban governance, social solidarity economy, urban commons, social movements

I confirm that I have read this submission and believe that I have an appropriate level of expertise to confirm that it is of an acceptable scientific standard, however I have significant reservations, as outlined above.

# Response

**Published:** 2024-12-19

**Authors:** Colm Stockdale

**Affiliations:** Department of Economic and Regional Development, Panteion University of Social and Political Sciences, Athens, Attica, Greece

Thank you for the interesting comments.

 1. The urban-rural comparisons have been incorporated in the conclusion, but not in the discussion, which does reflect the differences between northern and southern Europe. The research was conducted in a rural context and there was no intention, and subsequently, no data was gathered on the urban level. As such an urban-rural comparison cannot be made. However, we have reflected on what a rural-urban comparison might mean for future research in the conclusion section.  

 2. The manuscript’s main goal is to provide a novel theoretical framework to reconceptualize SI processes and their transformative potential, which is examined through CWS. Therefore, the main conceptual and analytical concepts are assemblage thinking and post-capitalist thinking (Diverse and Community Economies and Wright’s framework). While the suggested amendment would allow a possibly clearer explanation, the combination of both assemblage thinking and post-capitalist thinking are required to enable a re-conceptualization of SI processes and their transformative processes. 

 The text has been re-worked to now include a section (section 3) for the conceptual framework, for it to be clearer for the reader. Moreover, the methodology section has been reworked to include assemblage thinking also as a methodological device. 

 3. The main contribution of the manuscript is to provide a theoretical framework that re-conceptualizes SI processes and allows us to understand their transformative potential. Moreover, the first reviewer commented “using the Wright types of utopian change as steps toward a new society is promising.” We have used Wright’s tripartite distinction of ruptural/symbiotic/ interstitial transformation to support our arguments and in particular, to unpack the different transformative practices (and strategies) a CWS might have inside and outside of the organisation. We also believe that this adds on the DCE framework and can provide a new understanding of the different community economies and their symbiotic or interstitial practices with/against the market and the state. 

 4. Thank you for the insightful comment. This has been worked in to the main text in the conclusion, regarding the contribution of the framework other than the framework itself. Particularly the last paragraphs brings in some of these reflections, for example how we may re-consider rural and urban when it comes to transformations. The limitations of gatekeepers, and the rhizomatic character of the CWS assemblages has also been expanded upon in the beginning of the discussion.

# Reviewer report: Reviewer response for version 1

**DOI:** 10.21956/openreseurope.19462.r44825

**Published:** 2024-10-17

**Authors:** Gary Bosworth

**Affiliations:** 1 Northumbria University, Newcastle upon Tyne, England, UK

This is a very interesting paper investigating the social innovation that is emerging within the CWS sector. The attempt to apply Assemblages and Diverse and Community Economies theories is quite ambitious and I would like to see a clearer aim and set of conclusions that identify either: (i) how these theories help to generate new understanding about CWS or (ii) how the research advances these theories by this new application.

 Moving to a few more specific points, the final paragraph of the introduction could benefit from a slightly clearer aim and/or justification of why you landed on social innovation here. The definition is clear, but is coworking really SI?  Presumably there is at least some aspect of commercial innovation going on here if the venues are to be economically viable. Fundamentally, coworking is a service that people consume for personal benefit so I think we need to be careful when discussing the social innovation aspects of CWS.

 I like the details of the case study areas – although note the need for a capital G on Greece in the subheading.  I would like just a little more detail on the methods though – perhaps a table summarizing the characteristics of the 30 interviewees and I am also curious how you achieved 3,000 hours of participant observation by spending 1 month in each location?!

 I am interested that you say the CWS “acts as a place where different people who would not normally cross paths can meet” – I have often wondered just how much mixing occurs in these places.  It would be good if you could elaborate – I see the multiple user groups, but do they really mix?  And then, in terms of Social Innovation, you can start digging more deeply into the reconfiguration of networks.  I also like that part of the rationale for participating is to collectively address weak infrastructure etc so, although I would still argue that is an example of transactional consumption, it is at least more cooperative in the way that it requires a critical mass of users to bring about any impact.  This first section of the findings has only one quotation from the primary research, so perhaps adding a few more would be helpful.

 The final conclusions, although supported by the research, just feel a little underwhelming. This appears to be the main claim:

*“The findings reveal that CWS play a significant role in addressing social needs and facilitating grassroots SI practices, creating affective relations that contribute to the transformation of individual to collective subjectivities. CWS seem to provide a site where the unmet needs of the local people are negotiated, redefined and are covered in a co-creative and collective manner. This is happening in a DCE framework, where affective labour mostly performs.”*

 It would help the reader to see a clearer re-interpretation of the earlier theories or a summary of how those theories have deepened our understanding of CWS in order to address the initial aims of the paper. Perhaps it would help to state the more applied and more theoretical conclusions separately before a final paragraph that focuses back in on  the SI processes that are developing within CWS and their wider communities?

 A final very specific point, on P1 regarding this quotation: “Recent research suggests that 66% of CWS are located in urban regions, while the rest (34%) are in intermediate or rural regions”

 For this statement, it would probably be worth noting that it is difficult to collate accurate data for more rural areas given the variety of spaces that might class as CWS.   Also, you should state the geography to which the comment relate – is that Europe?

 Essentially, in addition to the minor typographical, methodological and explanation details, I would just like to see a few lines in the conclusion that speak back to a clearer aim which would help to frame the paper and make it easier for the reader to navigate the complexity of Assemblages, Social Innovation and Diverse and Community Economies.

If applicable, is the statistical analysis and its interpretation appropriate?

Not applicable

Is the study design appropriate and is the work technically sound?

Yes

Is the work clearly and accurately presented and does it engage with the current literature?

Yes

Are the conclusions drawn adequately supported by the results?

Yes

Are sufficient details of methods and analysis provided to allow replication by others?

Partly

Are all the source data and materials underlying the results available?

Partly

Reviewer Expertise:

NA

I confirm that I have read this submission and believe that I have an appropriate level of expertise to confirm that it is of an acceptable scientific standard, however I have significant reservations, as outlined above.

# Response

**Published:** 2024-12-19

**Authors:** Colm Stockdale

**Affiliations:** Department of Economic and Regional Development, Panteion University of Social and Political Sciences, Athens, Attica, Greece

**Regarding the first comment, the introduction overall has been amended to incorporate this and comments from other reviewers. The first paragraphs introduce the polycrisis and the rationale behind the necessity for societal transformations, of which social innovation is one of many means of achieving this.** **Coworking is not SI, and the paper does not state so. The CWS engage in SI processes (among many other activities, such as commercial innovation). Moreover, this paper is not about coworking spaces, it is about collaborative workspaces that includes a diversity of organizational forms. Through the framework of diversity and community economies we acknowledge in the discussion there are aspects of commercial innovation occurring. This is particularly clear in the case of WGH rather than the municipally funded CUA, as the former needs to trade on the market and write funding applications to remain economically viable, commercial innovation occurs in actions such as their boat tours or their festival. However, this is not the focus of the paper, the focus being what is the potential of these practices for post-capitalist transformations? Moreover, through the analytical framework of assemblage thinking, we go beyond spatial conceptions of CWS as bounded entities, and view them as CWS assemblages, which is clear from the interactions and actions both CWS have with their local communities.**

**Regarding the methods, a table has been added to section 4.1, and the typo of 3,000 hours has been corrected to 300.** **Regarding the comment ‘how much mixing occurs in these places’ this has been further elaborated upon. Particularly in section 5.1.2. , comparing the two cases under how much key individuals take part in other areas and actions of the CWS. Additional quotes have been added to the first section of the findings.** **The final conclusion has been changed, which now offers a summary of the findings, followed by a short and clear explanation of how the theories have contributed to thinking about social innovation and collaborative workspaces.** **The CORAL-ITN survey and the methods used for obtaining the relevant data are now acknowledged in the text, as well as the limitations of that methodology (specific keywords etc).** **The third paragraph in the conclusion, now states the main aim and contribution of the paper, and how they enrich both CWS and SI theory.  **   

# Reviewer report: Reviewer response for version 1

**DOI:** 10.21956/openreseurope.19462.r44829

**Published:** 2024-10-16

**Authors:** Bettina Bock

**Affiliations:** 1 University of Groningen, Groningen, The Netherlands; 2 Social Sciences, Wageningen University & Research Rural Sociology Group, Wageningen, Gelderland, Netherlands Antilles

This paper discusses the results of a study that compares the functioning of two rural collaborative workspaces in Greece and Austria, particularly the insights gained through elaborating a novel theoretical framework created for this purpose. The work is well embedded in current literature on workspaces, social innovation in rural areas, as well as recent theories aiming to understand social innovation and the coming into existence of new community-oriented economies. The work is clearly and accurately presented. It includes relevant information on the study design, which is both appropriate and technically sound. The paper provides sufficient insight into the methods used and provides access to the data collected. This should allow for replication with the remark that qualitative analysis is notoriously difficult to replicate. The paper does not refer to any statistical analysis. The conclusions drawn are supported by the results.

 The paper is particularly interesting as it tests the insights that may be gained when departing from a novel theoretical perspective. It does present empiric results, but more engaging is the potential that the new theoretical frameworks offer for understanding collective workspaces and other socially innovative initiatives in what transformation they promote and what could be done to support their ability to do so. In my view, the attempt to go beyond usual social innovation studies in which we focus on the results achieved, this study allows us to better understand the innovative elements of the process of change while keeping track of the specific context in which the innovative workspaces operate. I was engaged by the interlinkages made with social innovation literature, community economy and utopian thinking as altogether, they allow us to tease out what is really innovative and transformative. Using assemblage theory helps us to understand how social innovation can be understood in context and in interaction with the ‘outside world’. In building this new framework, they manage to reveal where the workspaces start to make a difference by changing social dynamics at the collective level as well as among individuals who start to think differently thereby considering themselves as parts of collectives. Also, using the Wright types of utopian change as steps toward a new society is promising.  

 Overall, I think the paper is almost ready for indexing. As a reader, I would have liked to see a clear formulation of research questions, clarifying also that the main purpose is a theoretical one. The study is presented as a comparative one, and in the result sections, both cases are presented. There is also some reference to comparing, however, focusing mainly on the correspondences. Certainly, when arriving at the point of the transformative potential, it would be interesting to learn more about the differences between the cases. One could imagine that the transformative potential is higher in the case of Greece, given the more peripheral context and higher attractiveness of social economies/commons, but maybe the difference in potential is also related to differences between what the workspaces are and do. We can, after all, learn a lot about the novel theoretical framework where it identifies differences or where it fits better given the context.

If applicable, is the statistical analysis and its interpretation appropriate?

Not applicable

Is the study design appropriate and is the work technically sound?

Yes

Is the work clearly and accurately presented and does it engage with the current literature?

Yes

Are the conclusions drawn adequately supported by the results?

Yes

Are sufficient details of methods and analysis provided to allow replication by others?

Yes

Are all the source data and materials underlying the results available?

Yes

Reviewer Expertise:

rural development, social innovation, peripheralisation, community initiatives

I confirm that I have read this submission and believe that I have an appropriate level of expertise to confirm that it is of an acceptable scientific standard, however I have significant reservations, as outlined above.

# Response

**Published:** 2024-12-19

**Authors:** Colm Stockdale

**Affiliations:** Department of Economic and Regional Development, Panteion University of Social and Political Sciences, Athens, Attica, Greece

Thank you for the comments and feedback. The research questions are now stated at the end of section 2.2. at the end of the literature review. 

 Regarding the comparison of the cases and their transformative potential, it is a very constructive comment. This was integrated into the beginning of the discussion section.
